# Beyond Foxp3 and TSDR: a dual epigenetic lock framework for treg stability and plasticity

**DOI:** 10.3389/fimmu.2026.1952451

**Published:** 2026-09-22

**Authors:** Feng Wang, Haiying Yang, Zhiguo Xu, jingxian Song, Zijun Wang, Le Wang

**Affiliations:** 1 Huzhou Central Blood Station, Huzhou, China; 2 Huzhou College of Life and Health, Huzhou, China; 3 Zhejiang Chinese Medical University, Hangzhou, China

**Keywords:** CAR-Treg engineering, epigenetic plasticity, ex-Treg conversion, regulatory T cells (Tregs), super-enhancers, TET dioxygenases, TSDR demethylation

## Abstract

Regulatory T cells (Tregs) maintain immune homeostasis by balancing lineage stability with functional adaptability. Although Foxp3 is central to Treg identity, Foxp3 expression alone does not fully explain the long-term maintenance of suppressive function or the capacity of Tregs to adapt to diverse tissue environments. Similarly, Foxp3 TSDR demethylation provides a durable epigenetic imprint but does not account for the dynamic regulation of broader Treg functional programs. Here, we propose the Dual Epigenetic Lock framework as a conceptual and testable model in which two complementary epigenetic layers contribute to Treg state regulation. The first lock consists of stable TSDR demethylation, which preserves lineage-associated Foxp3 expression competence. The second lock is proposed to involve Treg-associated super-enhancer networks that may stabilize suppressive transcriptional programs, regulate environmental adaptation, and constrain inappropriate inflammatory activation. This framework suggests that Treg stability emerges from the integration of durable lineage memory and dynamic functional regulation rather than from a single molecular determinant. Disruption of this balance may contribute to impaired immune tolerance in autoimmune diseases, whereas excessive reinforcement of suppressive regulatory programs may support tumor immune evasion. However, the extent to which these two epigenetic layers are mechanistically coupled remains unresolved. Emerging approaches including single-cell multi-omics, spatial epigenomics, and targeted epigenome engineering provide opportunities to test whether the dual-lock architecture predicts Treg state transitions and therapeutic responsiveness. This framework also provides a conceptual basis for two complementary therapeutic strategies: reinforcing Treg stability for immune tolerance and selectively modulating tumor-associated Treg programs for cancer immunotherapy.

## 1 Introduction: Treg stability as a core challenge in immune homeostasis

### 1.1 The therapeutic paradox of Treg stability

Foxp3^+^ regulatory T cells (Tregs) are essential for immune homeostasis. Their absence causes severe systemic autoimmunity in patients with IPEX syndrome and Foxp3-deficient mice (1–3). Conversely, tumors exploit this lineage through RBPJ–NCOR and HDAC3 complexes to establish immunosuppression (4). This dual role creates a therapeutic paradox: Treg activity should be reinforced in autoimmune disorders but restrained in cancer.

Maintaining Treg stability while preserving functional adaptability lies at the heart of this paradox. The traditional view of Foxp3 as an irreversible lineage commitment has been challenged by evidence that some Tregs lose Foxp3 and acquire pro-inflammatory features, including IFN-γ production, generating ex-Treg cells (5–7). Fate-mapping studies show exFOXP3 cells accumulate in inflammatory settings such as the central nervous system during autoimmunity (8). This phenomenon may not be a simple lineage failure. Instead, it may reflect a dynamic balance between immune tolerance and adaptation to inflammation. This has important therapeutic implications.

Recent conditional Foxp3 degradation studies suggest that transient Foxp3 downregulation does not necessarily mean irreversible lineage loss (9). Treg stability therefore should not be viewed as a binary state, but as a dynamic equilibrium shaped by durable lineage-associated mechanisms and context-dependent regulatory adaptation.

### 1.2 Limitations of existing models and the need for an integrative framework

Two conceptual frameworks have shaped our understanding of Treg identity. The Foxp3-centric model established Foxp3 as a lineage-defining transcription factor. However, Foxp3 expression alone does not fully explain the maintenance of suppressive capacity, tissue adaptation, or inflammatory specialization. Tregs can transiently reduce Foxp3 without permanently losing functional identity, and Foxp3 deficiency does not invariably cause immediate lineage collapse (5, 10). Thus, Foxp3 represents a critical but incomplete determinant of Treg stability.

The TSDR-centric model provided a molecular explanation for lineage heritability. Stable demethylation of the Foxp3 Treg-specific demethylated region (TSDR) functions as an epigenetic imprint that sustains Foxp3 expression competence through cell division, even when Foxp3 transcription fluctuates (11, 12). However, TSDR demethylation alone does not explain how Tregs dynamically reorganise their enhancer landscape during tissue adaptation, inflammatory responses, or functional specialization. Nor does it fully account for the extensive functional heterogeneity among Treg populations across different physiological contexts.

Neither framework alone sufficiently explains the tunable equilibrium between lineage fidelity and functional plasticity observed *in vivo*. To address this gap, we propose the Dual Epigenetic Lock framework. Treg stability depends on two regulatory layers. The first is a durable epigenetic imprint that keeps Foxp3 expression competent. The second is a dynamic network that coordinates suppressive programs and limits inappropriate inflammatory gene activation.

To anchor this framework in measurable terms, we define Treg stability along three operational dimensions. Epigenetic lineage stability refers to the maintenance of TSDR hypomethylation, which preserves Foxp3 re-expression potential. This dimension is primarily governed by the first lock. Phenotypic suppressive stability refers to the sustained expression of key inhibitory molecules (such as CTLA-4 and IL-10) and the restriction of inflammatory cytokine production. This dimension is dynamically regulated by the second lock. Environmental resilience refers to the capacity to maintain both dimensions in the face of inflammatory or metabolic stress, reflecting the integrated function of both locks. Within this framework, Treg “plasticity” represents functional fluctuations within dimensions 2 and 3, while “instability” specifically denotes erosion of dimension 1 leading to irreversible lineage loss.

### 1.3 The dual epigenetic lock: a conceptual framework and review scope

This review systematically examines the molecular basis and biological implications of the dual epigenetic lock model. The first lock is stable demethylation of the TSDR within the Foxp3 locus, which provides durable Foxp3 expression competence and lineage stability (12–14). The second lock comprises a dynamic network of Treg-associated super-enhancers (Treg-SEs) that coordinate transcriptional programs supporting suppressive function, environmental responsiveness, and adaptive specialization. Through CTCF/cohesin-mediated chromatin organization, these enhancer networks contribute to higher-order regulatory control of Treg signature genes while potentially constraining inappropriate activation of inflammatory loci such as Ifng (15–17).

The term “lock” denotes a stabilizing regulatory function, not an irreversible state. Unlike the first lock, the second lock remains highly responsive to environmental signals and can be remodeled according to tissue context and inflammatory conditions. TET dioxygenases are candidate molecular coordinators linking TSDR demethylation with enhancer-associated chromatin regulation, as suggested by Tet2/Tet3 loss-of-function models (18, 19). Whether TET-mediated regulation directly integrates these two epigenetic layers in mature Tregs requires further investigation.

This review examines the molecular architecture of the dual epigenetic lock framework. We discuss how inflammatory signals such as IL-6/STAT3 and IL-12/STAT4, together with metabolic disturbances, may disrupt regulatory stability through pathways involving DNMTs, HDACs, and chromatin remodeling. We also discuss emerging technologies, including single-cell multi-omics and spatial epigenomics, that enable investigation of Treg regulatory states at unprecedented resolution (20, 21). Finally, we explore two complementary therapeutic directions: reinforcing Treg stability to generate controllably durable CAR-Treg products for autoimmune diseases, and selectively modulating suppressive Treg programs to enhance anti-tumor immunity.

We should view Treg stability as a tunable system, not a fixed property. This review builds on that view. It offers an integrative framework. The framework helps us understand how epigenetic mechanisms balance tolerance, adaptation, and therapy.

## 2 The first lock: TSDR, a durable epigenetic imprint of Treg lineage stability

The Treg-specific demethylated region (TSDR) is a highly conserved, CpG-rich regulatory element located within the first intron of the *Foxp3* locus. Stable demethylation of this region represents a defining epigenetic feature of thymus-derived regulatory T cells (tTregs) and establishes a chromatin state that supports sustained Foxp3 expression across cell divisions (11, 13). Comparative analyses of *in vitro*-induced Tregs (iTregs) have shown that TSDR demethylation is frequently incomplete in these cells, which may contribute to reduced Foxp3 stability under inflammatory conditions. This contrasts with the more stable Foxp3 expression competence associated with deeper TSDR demethylation in tTregs (22, 23).

The TSDR does not act as an isolated molecular switch. Instead, it is a durable epigenetic imprint that keeps the *Foxp3* locus transcriptionally competent. This distinction is important because TSDR status reflects a lineage-associated chromatin state but does not, by itself, fully define the functional diversity or environmental responsiveness of Tregs. Within the dual epigenetic lock framework, the TSDR constitutes the first regulatory layer by maintaining lineage fidelity and providing a stable foundation upon which additional regulatory mechanisms can operate.

### 2.1 The regulatory blueprint of the Foxp3 locus

The Foxp3 locus includes its promoter and three conserved non-coding sequences (CNS1–3). CNS1 and CNS3 contribute to peripheral and thymic Treg development, respectively. CNS2 (TSDR) is a CpG-rich region in the Foxp3 first intron. It exhibits the most prominent epigenetic difference between Tregs and conventional T cells. Floess et al. confirmed its functional role by showing TSDR demethylation in Tregs versus methylation in Tconv via systematic CpG analysis (11). Ohkura et al. later showed that TSDR demethylation develops alongside, but is not simply dependent on, Foxp3 expression during thymic Treg differentiation (24). These findings established that epigenetic stabilization of the Foxp3 locus represents an additional regulatory layer beyond transcription factor expression alone.

Recent multi-omics analyses further support this view. Human tTregs exhibit greater chromatin accessibility and more extensive TSDR demethylation than iTregs generated *in vitro* (22). This epigenetic configuration maintains Foxp3 expression competence and contributes to long-term lineage stability (**Figure 1**). However, the TSDR should be viewed as a permissive regulatory element rather than a complete determinant of Treg identity. Additional chromatin-based mechanisms are required to establish and maintain the broader Treg transcriptional program.

**Figure 1:**
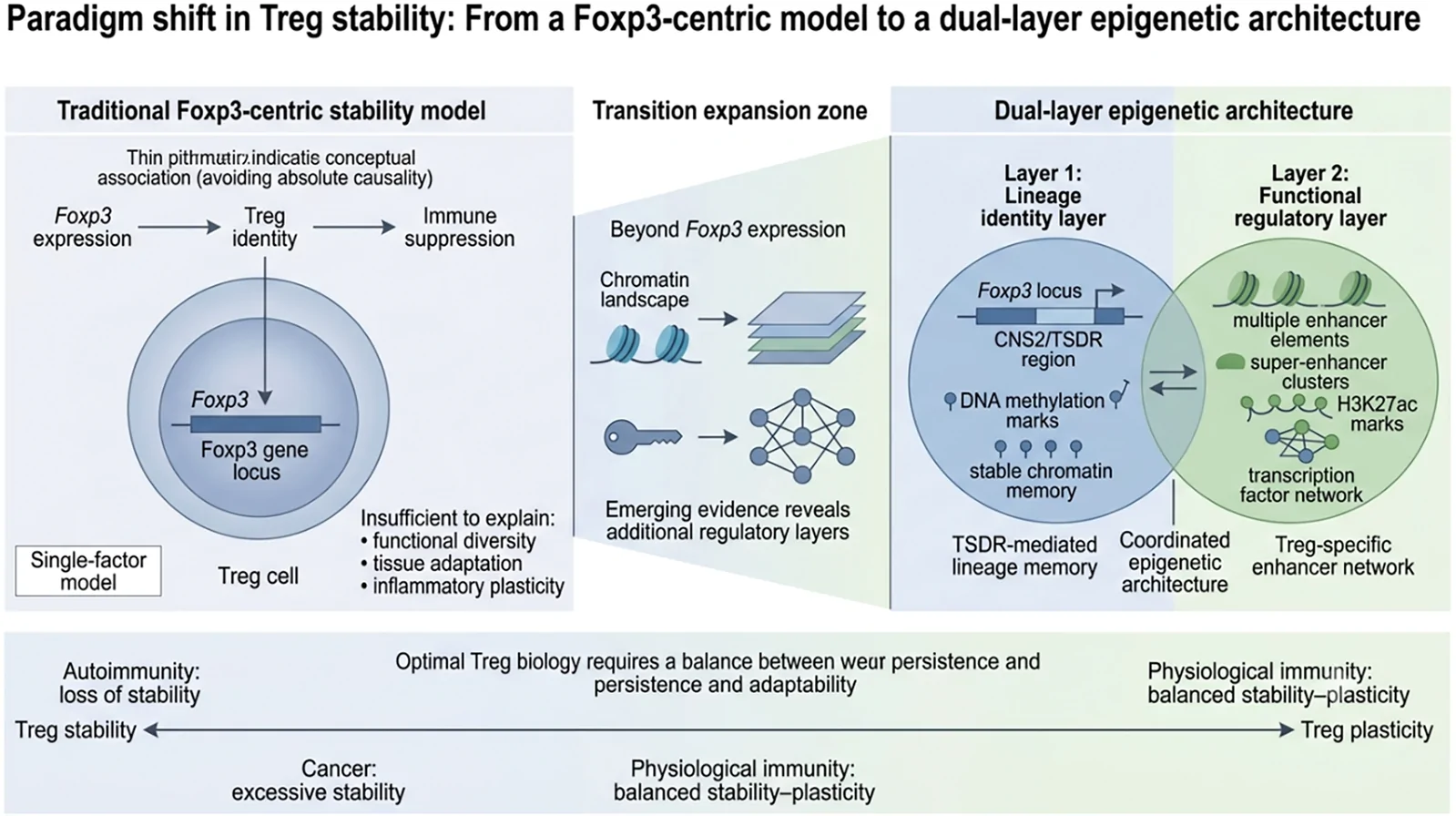
Detailed structural map of the Foxp3 locus and TSDR. The TSDR is not an isolated methylation switch. Rather, it sits as a central node within a regulatory network composed of four conserved non-coding sequences (CNS1–3 and the promoter) embedded in the *Foxp3* locus. The principal transcription factor binding profiles across these elements are annotated. The magnified region displays the starkly contrasting CpG modification status at the TSDR—fully demethylated in Tregs (○) versus heavily methylated in conventional T cells (●). This divergence constitutes the first passive imprint of Treg lineage identity.

The distinction between thymic and peripheral Treg origin should not, however, be interpreted as an immutable epigenetic dichotomy. Although tTregs generally exhibit more extensive TSDR demethylation (25, 26), peripheral Tregs may undergo further epigenetic maturation after prolonged antigen exposure or sustained tissue adaptation (27). Thus, under appropriate conditions, some pTregs may acquire a more consolidated TSDR state that approaches that of mature tTregs. Such convergence would not necessarily imply complete convergence of the two populations. TSDR demethylation primarily reflects lineage-associated Foxp3 competence, whereas the broader Treg super-enhancer landscape may remain shaped by developmental history (25), antigenic experience, and tissue-specific signals. Within the dual-lock framework, therefore, developmental origin may influence the starting configuration of Lock 1 without rigidly determining its subsequent state (28).

We define Lock 1 as a TSDR-centric module. This choice requires justification. Treg identity is accompanied by genome-wide DNA hypomethylation at multiple loci (26). However, TSDR is unique in three respects. Its demethylation status is the most robust correlate of heritable Foxp3 expression competence across cell divisions (29). It distinguishes committed tTregs from transient Foxp3+ activated Tconv cells (30). Its remethylation marks irreversible lineage loss in ex-Tregs. Other hypomethylated loci do not share this lineage-restricted pattern.

Two human-specific caveats require attention. First, FOXP3 is X-linked (31). In females, TSDR methylation assays must account for X-chromosome inactivation skewing (32). Mosaic methylation patterns may reflect XCI status rather than Treg lineage heterogeneity. Second, activated human Tconv cells can transiently upregulate FOXP3 without TSDR demethylation (33). This dissociation reinforces a central premise of our framework. Foxp3 protein alone does not define stable Treg identity. TSDR demethylation marks the committed lineage. These considerations underscore why measuring both TSDR methylation and Foxp3 expression is essential in human studies.

### 2.2 The enzymatic logic of demethylation

TSDR demethylation is mediated by ten-eleven translocation (TET) dioxygenases, which iteratively oxidise 5-methylcytosine (5mC) to 5-hydroxymethylcytosine (5hmC), 5-formylcytosine (5fC), and 5-carboxylcytosine (5caC). These intermediates are processed by TDG-dependent base excision repair to restore unmodified cytosine (11, 34). This process begins during thymic Treg development and establishes stable Treg populations (22).

Environmental and metabolic signals modulate TET activity at the Foxp3 locus. Vitamin C enhances TET2-dependent demethylation at Foxp3 CNS2 and promotes Treg differentiation (35). Hypoxia increases TET2 and TET3 expression and enhances Foxp3 demethylation in iTregs (36). Hydrogen sulfide also regulates TET1/2 activity, linking metabolic state to epigenetic maintenance of Treg identity (37). TET activity may also be influenced by cell-surface metabolic sensing. The CD147–CD98 transport-associated axis has been implicated in coupling extracellular nutrient and metabolite availability to intracellular metabolic states and, potentially, to α-ketoglutarate-dependent dioxygenase activity (38, 39). In Tregs, such a pathway could provide an upstream connection between membrane-proximal metabolic sensing and the biochemical environment required for TET-mediated DNA demethylation (40). However, whether CD147–CD98 directly regulates TET activity at the Foxp3 locus in primary Tregs remains insufficiently established. We therefore regard this pathway as a plausible upstream modifier of the first lock rather than as a demonstrated Treg-specific regulatory mechanism.

The extent and stability of TSDR demethylation depend strongly on Treg developmental origin. tTregs progressively acquire stable TSDR demethylation during thymic maturation, reaching a consolidated epigenetic state after full lineage maturation (13, 41). In contrast, peripheral iTregs frequently display incomplete demethylation and substantial inter-individual variation, which may contribute to differences in Foxp3 stability under inflammatory conditions (11, 23). This distinction has important implications for therapeutic applications, including CAR-Treg manufacturing, in which the cellular source may influence the durability and functional stability of engineered Tregs.

### 2.3 TSDR demethylation provides lineage stability but does not fully define Treg function

Consistent with the principle that Foxp3 competence does not equate to full functional programming (Section 1.2), targeted TSDR demethylation alone is insufficient to establish a stable Treg transcriptional programme. For example, targeting CNS2 with dCas9-TET1CD only caused partial TSDR demethylation. This was not enough to sustain Foxp3 expression. In contrast, activating the promoter with dCas9-p300HAT maintained Foxp3 activity more effectively (42). Similarly, Arroyo-Olarte et al. demonstrated that targeted FOXP3-TSDR demethylation enhanced Foxp3 expression and suppressive activity in iTregs but did not fully restore the transcriptomic profile characteristic of mature Tregs (43). These observations reinforce that TSDR demethylation supports Foxp3 competence but does not independently encode the full Treg identity program.

The broader role of TET enzymes further illustrates this principle. Tet2/Tet3 double-knockout models demonstrate that TET deficiency results not only in TSDR remethylation and Foxp3 destabilization but also in aberrant inflammatory programs, including increased IL-17 expression and acquisition of pro-inflammatory features by Tregs (18, 19). These broad functional abnormalities cannot be attributed solely to altered TSDR methylation. Instead, they suggest that TET enzymes contribute to a wider chromatin regulatory network that coordinates multiple aspects of Treg identity and function.

The TSDR alone, however, does not account for the functional heterogeneity and environmental responsiveness of Tregs. This brings us to the second lock: the Treg-associated super-enhancer network. Evidence supporting the first lock model is stratified according to confidence level in **Table 1**.

**Table 1:** Evidence stratification supporting the first epigenetic lock (TSDR) model.

Model component	Biological claim	Key supporting data	Evidence level	Critical gaps/future directions
TSDR Demethylation & Foxp3 Stability	Complete TSDR demethylation is a hallmark of tTregs and supports heritable Foxp3 expression	Near-complete TSDR demethylation in tTregs vs. heavy methylation in Tconv (11) TSDR demethylation occurs independently of Foxp3 expression during thymic development (24) Human tTregs exhibit more complete TSDR demethylation than iTregs (22)	Established	-–
tTreg vs. iTreg TSDR Differences	Incomplete TSDR demethylation in iTregs renders them susceptible to Foxp3 loss under inflammatory conditions	iTregs show incomplete TSDR demethylation and Foxp3 instability upon cytokine exposure (22, 44) tTregs maintain stable Foxp3 expression due to fully demethylated TSDR	Established	Direct *in vivo* comparison of tTreg and iTreg TSDR dynamics during inflammation is limited. Whether prolonged antigen exposure permits substantial TSDR maturation in pTregs remains incompletely defined.
TSDR Necessity but Insufficiency	TSDR demethylation is necessary but not sufficient for full Treg functional programme	dCas9-TET1CD-mediated TSDR demethylation alone induces only partial Foxp3 expression (42) dCas9-p300HAT co-targeting of promoter required for persistent activity (42) Targeted FOXP3-TSDR demethylation fails to restore full Treg transcriptome (43)	Established	Whether TSDR demethylation combined with specific TF activation can fully reconstitute Treg identity remains unknown
TET Catalytic Role in TSDR Maintenance	TET2/3 are the core catalytic enzymes maintaining TSDR demethylation	Tet2/Tet3 DKO leads to TSDR remethylation and Foxp3 loss (18, 19) Vitamin C enhances TET2-dependent TSDR demethylation (35) H_2_S boosts TET1/2 activity (37)	Established	-–
TSDR as a “Lineage Memory Layer”	TSDR demethylation provides a heritable chromatin configuration enabling the Foxp3 locus to respond to activating signals	TSDR demethylation persists through cell division independently of Foxp3 expression (45) TSDR acts as a “passive imprint” rather than a deterministic lock (22)	Emerging	Whether TSDR demethylation directly transmits information to Treg-SEs via a reader protein remains unknown
TSDR-Treg-SE Synergy	TSDR supplies Foxp3 protein; Treg-SEs organize functional programme; both layers cooperate	Foxp3 binds enhancer-promoter loops and reinforces H3K27ac at Treg-SEs(c15) Foxp3 organizes Treg three-dimensional genome architecture (16, 17)	Emerging	Whether TSDR methylation status actively instructs SE activity or is merely permissive is unresolved
Metabolic-Epigenetic Coupling at TSDR	Microenvironmental metabolites modulate TET activity and TSDR methylation status	Vitamin C enhances TET2 activity (35) Hypoxia upregulates TET2/3 and enhances demethylation (46) H_2_S links metabolic status to epigenetic homeostasis (37)	Emerging	Therapeutic window for metabolic intervention remains empirical

Evidence Level Definitions: Established, supported by multiple independent lines of evidence including *in vivo* genetic models and human data; Emerging, supported by experimental data but with limited *in vivo* validation or unresolved mechanistic details; Hypothesis, inferred from indirect evidence or non-Treg systems; direct experimental validation in Tregs is pending.

## 3 The second lock: the Treg-associated super-enhancer network—a dynamic regulatory hub

TSDR demethylation gives a durable epigenetic base for Foxp3 competence. The Treg-SE network is the second lock. It is a dynamic layer that coordinates Treg functions. It also helps control inflammatory gene expression. Unlike the first lock, which primarily reflects a lineage-associated epigenetic imprint, the second lock integrates transcription factor activity, enhancer accessibility, and three-dimensional chromatin organization to support context-dependent Treg adaptation.

Treg-SE networks are not fixed regulatory modules. Instead, they respond strongly to developmental cues, cytokine signals, and metabolic conditions. Through coordinated enhancer activity and chromatin remodeling, these networks help maintain suppressive programs while allowing Tregs to adapt to distinct tissue environments. Thus, the second lock provides the functional flexibility required for Tregs to preserve immune tolerance under dynamic physiological and pathological conditions.

### 3.1 Discovery of super-enhancers and their unique status in Tregs

Super-enhancers (SEs) are large regulatory domains that coordinate cell-state-specific transcriptional programs. Kitagawa et al. mapped SE landscapes in Tregs. They showed that key Treg genes (Foxp3, Ctla4, Il2ra, Ikzf2) associate with large SE clusters. Formation of these clusters depends on SATB1, a chromatin organizer (47, 48). Conditional Satb1 deletion disrupts Treg-SE formation and alters the balance between suppressive and effector programs, promoting effector-like features in thymic Tregs. Satb1-deficient mice develop severe autoimmune pathology, supporting the concept that Treg-SE networks represent an important component of the second lock.

Consistent with the observation that Foxp3 expression alone is insufficient (Section 1.2), the Treg-SE network provides an additional regulatory layer that complements TSDR-mediated stability. Sustained regulatory function requires coordinated control of broader transcriptional programs embedded within enhancer landscapes.

### 3.2 Core composition: a Foxp3-centered transcriptional regulatory network

Foxp3 contributes to the establishment and maintenance of Treg-SE-associated regulatory programs. Foxp3 HiChIP studies show that Foxp3 reinforces enhancer-promoter interactions at key Treg loci (Foxp3, Ctla4, Il2ra) by promoting active chromatin states (16). These findings extend the role of Foxp3 beyond direct transcriptional regulation and implicate it in the organization of higher-order chromatin architecture in Tregs.

Similar SE-associated regulatory features have been identified in human Tregs. H3K27ac ChIP-seq profiling of human CD4^+^CD25^hiCD127^lo Tregs revealed prominent SE clusters associated with the *FOXP3*, *CTLA4*, and *IL2RA* loci (49). These observations suggest that enhancer-based regulatory mechanisms are conserved across species, while remaining responsive to cell type and environmental context.

Cytokine signaling further modulates Treg-SE activity. IL-2-induced STAT5 signaling occupies multiple regions within Treg-SE landscapes and contributes to the activation of Treg-associated transcriptional programs (50). Importantly, the temporal relationship between enhancer activation and DNA demethylation appears more complex than a simple sequential model. Li et al. demonstrated that CBP/p300-mediated histone acetylation at the *Foxp3* promoter precedes TET-dependent DNA demethylation, suggesting that components of the second lock can become active before full establishment of the first lock (51).

Additional regulatory factors fine-tune SE activity according to cellular context. BACH2 maintains repressive enhancer configurations in resting Tregs and limits premature effector differentiation, whereas Blimp1 promotes activation of effector-associated transcriptional modules (52). Furthermore, genome-wide CRISPR screening has identified chromatin remodeling factors, including components of the BAF/SWI-SNF complexes, as important regulators of Treg phenotypic stability (53). Collectively, these findings indicate that the second lock represents a dynamic regulatory network rather than a static enhancer structure, integrating transcription factors, signaling pathways, and chromatin remodeling machinery.

### 3.3 Three-dimensional chromatin architecture and spatial insulation

Regulation of Treg-SE target genes is influenced by three-dimensional chromatin architecture involving CTCF- and cohesin-mediated interactions. Chromatin conformation studies, including HiChIP analyses, have shown that Treg-SEs establish spatial contacts with target promoters through enhancer–promoter loops that can be dynamically reorganized during Treg activation (15, 16). These interactions provide a structural basis for coordinated regulation of lineage-associated gene networks.

An additional feature of higher-order chromatin organization is the spatial restriction of inappropriate inflammatory gene activation. Muto et al. demonstrated that a CTCF-binding site contributes to the three-dimensional organization of the *Ifng* locus. In stable Tregs, the *Ifng* region is maintained within an insulated topologically associating domain (TAD) that is spatially separated from active chromatin domains containing Treg-associated regulatory elements (17).

How this insulation is altered during inflammatory conversion remains an important unresolved question. Potential mechanisms include IL-12/STAT4/T-bet-driven remodeling of CTCF occupancy (17, 54), changes in cohesin-mediated looping, or broader restructuring of inflammatory gene-associated chromatin domains (55). Determining whether these events directly drive ex-Treg conversion or represent downstream consequences of inflammatory activation will require further functional investigation.

### 3.4 Functional integration of the two locks

Stable TSDR demethylation establishes a permissive chromatin environment that supports sustained Foxp3 expression, whereas Treg-SE networks provide a dynamic regulatory framework for executing and adapting Treg functional programs. New Foxp3 protein binds to regulatory regions in Treg enhancer networks via its forkhead domain. It forms complexes with partners like NFAT and AP-1. This helps activate enhancers and connect promoters to enhancers (16).

CNS2 is not merely a passive imprint. It also functions as a dynamic regulatory element (56). It recruits transcription factors including CREB, Ets-1 and Foxp3 itself (57, 58). This places CNS2 at a physical interface between the two locks (26). Through recruited factors and chromatin remodelers, CNS2 can integrate signals from the Treg-SE network (25, 59). Its methylation status provides a permissive foundation for Foxp3 re-expression. This in turn enables sustained engagement of enhancer elements. This interpretation aligns with the reciprocal feedback model presented in **Figure 2** (58).This proposed relationship suggests mutual reinforcement. It is not a simple hierarchical pathway (**Figure 2**) (15, 16). Importantly, the extent to which these two regulatory layers are mechanistically coupled remains unresolved. Current evidence supports functional interaction between DNA methylation states, transcription factor networks, and enhancer landscapes (19), but does not yet establish a single integrated regulatory circuit.

**Figure 2:**
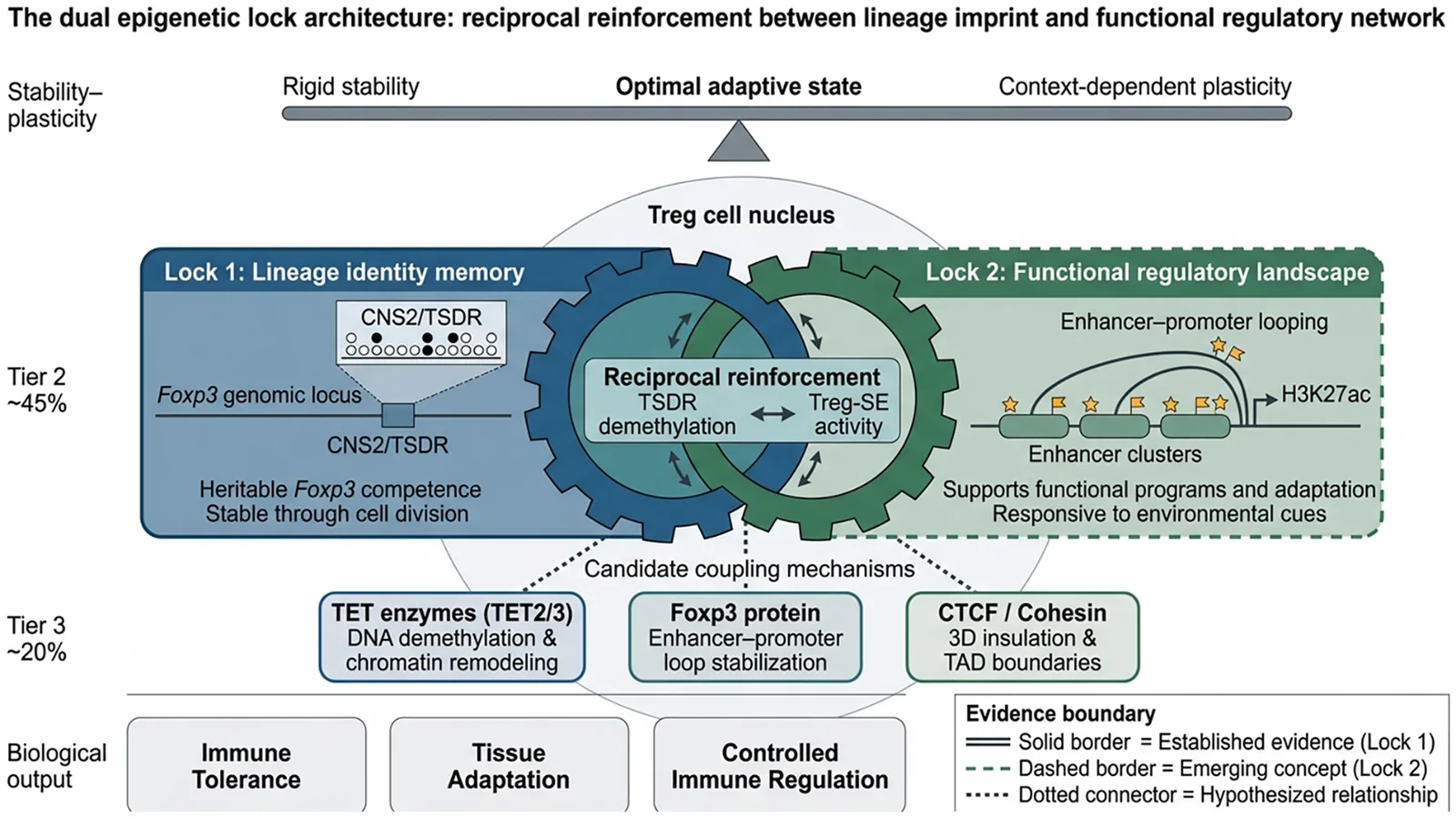
The dual epigenetic lock architecture: reciprocal reinforcement between lineage imprint and functional regulation. This image presents the conceptual framework of the dual epigenetic lock model, in which Treg stability emerges from the integration of two complementary regulatory layers depicted as structurally interlocked modules. Lock 1 (left; solid border, established evidence) represents the TSDR/CNS2 epigenetic imprint within the Foxp3 locus. Its stable demethylation provides heritable Foxp3 expression competence through cell division, thereby preserving lineage fidelity. Lock 2 (right; dashed border, emerging evidence) represents the Treg-associated super-enhancer (Treg-SE) network, comprising enhancer clusters, H3K27ac marks, and enhancer–promoter loops that coordinate suppressive transcriptional programs and enable tissue adaptation. The central interlocked region (blue–green gradient) denotes reciprocal reinforcement between the two locks, distinguishing this framework from linear hierarchy (Model A) or parallel independence (Model B) alternatives. Candidate molecular integrators—TET enzymes, Foxp3 protein, and CTCF/cohesin—are connected by dotted lines to indicate hypothesized coupling mechanisms rather than established pathways. The top stability–plasticity continuum emphasizes that the integrated state represents a dynamic equilibrium between rigid stability and context-dependent plasticity, rather than maximal stabilization. Biological outputs arising from this integrated regulation include immune tolerance, tissue adaptation, and controlled immune regulation.

TET enzymes represent candidate molecular links between these processes. Beyond their role in DNA demethylation, TET proteins may influence enhancer-associated chromatin regulation through interactions with transcriptional and chromatin-modifying complexes, including p300/CBP-associated mechanisms (60, 61). However, whether TET-mediated regulation directly coordinates TSDR demethylation with Treg-SE activity requires further experimental validation.

Thus, TSDR preserves Foxp3 competence, while Treg-SE networks translate that competence into functional adaptation. Neither layer alone is sufficient: stable lineage-associated regulation without functional execution may fail to achieve effective immune suppression, whereas functional adaptation without durable lineage control may increase the risk of inflammatory conversion.

Experimental strategies capable of integrating temporal epigenomics, perturbation-based approaches, and single-cell multi-omics will be essential for distinguishing these possibilities, as discussed in Section 9.

### 3.5 Alternative architectures for the two-lock relationship

Having defined the molecular basis of both locks, a central question emerges: what is the causal and logical relationship between the TSDR imprint and the Treg-SE network? We propose three alternative models (**Figure 3**). Model A (linear hierarchy): TSDR demethylation drives Foxp3 expression, which then passively induces Treg-SE assembly (11, 15, 55). Model B (parallel independent): the two locks are established and maintained independently by distinct upstream signals (24, 47, 50). Model C (reciprocal feedback, favoured here): TSDR provides a permissive environment for Foxp3 expression; Foxp3 participates in chromatin loop formation at Treg-SEs; and active Treg-SE signaling reinforces TSDR hypomethylation (11, 51, 55). Current indirect evidence favors Model C, but direct validation is lacking. We use these three models as analytical lenses to examine evidence from disease remodeling and therapeutic strategies (18, 19, 51).

**Figure 3:**
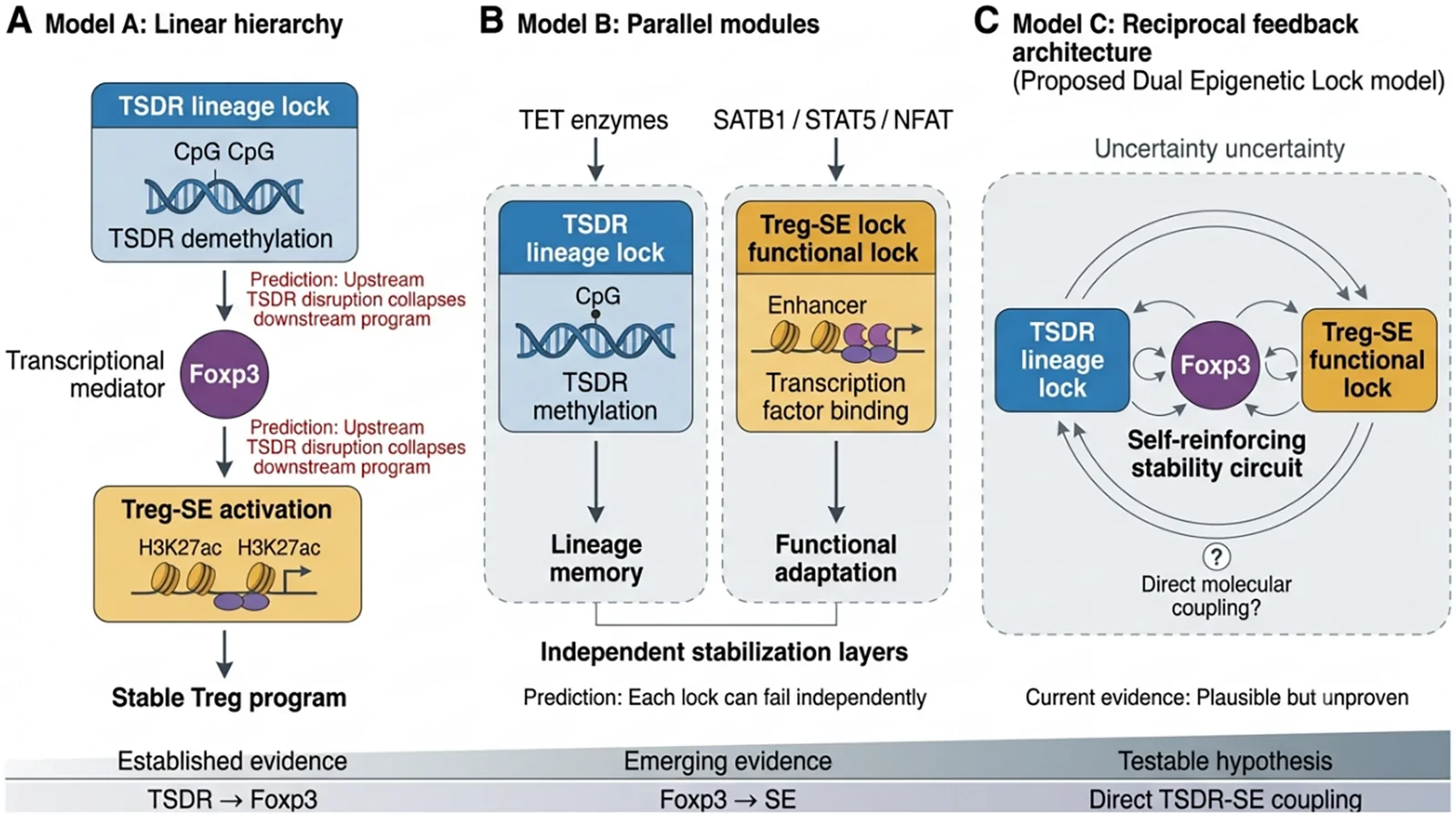
The relationship between TSDR-associated lineage memory and Treg-super enhancer (Treg-SE)-mediated functional regulation remains unresolved. Three possible models are illustrated: **(A)** Model A, a linear hierarchy in which TSDR stabilization promotes Foxp3 expression and downstream Treg-SE activation; **(B)** Model B, a parallel model in which TSDR and Treg-SEs function as partially independent regulatory modules; and **(C)** Model C, a reciprocal feedback model proposed by the Dual Epigenetic Lock framework, in which TSDR-mediated lineage memory and Foxp3-associated enhancer networks reinforce each other to stabilize Treg identity. Current evidence is consistent with, but does not yet prove, a feedback-coupled architecture. Direct experimental validation of temporal and causal interactions between these regulatory layers remains a major challenge.

## 4 TET enzymes: candidate molecular links between DNA demethylation and enhancer regulation

TET dioxygenases may link TSDR demethylation with Treg-SE regulation, but their direct coordination versus parallel function remains unresolved (18, 19). The emerging dual-lock framework places TET enzymes at a potential interface between stable lineage imprinting and dynamic transcriptional regulation (46). However, the evidence varies in strength. TET catalytic roles in TSDR demethylation are well established. Their roles in enhancer regulation are still mostly inferred from emerging data and mechanisms (34). Distinguishing these established and hypothetical functions is essential for accurately interpreting TET biology in Treg stability.

### 4.1 Established catalytic function and emerging hypotheses in chromatin regulation

TET2/3 have a clear catalytic role in TSDR demethylation (see Section 2.2 and **Table 2**). Separately, we can test whether TET proteins also affect the second lock. They might do so via non-catalytic binding to chromatin remodelers. In vascular smooth muscle cells, TET2 was found to co-immunoprecipitate with p300, and TET2 knockdown significantly reduced H3K27ac deposition at target gene promoters, indicating that TET2 is required for p300-dependent H3K27 acetylation (66, 67). TET-mediated demethylation may also recruit SWI/SNF chromatin remodeling complexes to regulatory regions (64). However, direct evidence for these non-catalytic functions in primary Tregs remains limited (Hypothesis; see **Table 2**). Whether TET-HAT interactions occur at Treg-associated super-enhancers, and whether they depend on TET catalytic activity, are unresolved.

**Table 2:** Evidence-stratified functions of TET enzymes and their therapeutic implications.

Functional dimension	Evidence level	Molecular mechanism	Manifestation in tregs	Therapeutic potential & challenges
Catalytic Demethylation Function	Established	Maintains TSDR in a fully demethylated state, ensuring heritable Foxp3 expression	Tet2/Tet3 DKO results in TSDR remethylation and Foxp3 loss (18, 19)	TET2 exhibits a Janus role in tumor immunity: inhibiting TET in Tregs may “unlock” them, but preserving TET in CD8^+^/NK cells is essential for anti-tumor function (19, 62, 63)
TET-HAT Interaction & Chromatin Remodeling	Emerging	TET-mediated demethylation is a prerequisite for SWI/SNF chromatin remodeling complex recruitment (34, 64)	Direct evidence in primary Tregs and functional consequence of TET-HAT interaction remain to be fully established	Combining with HDACi may restore Treg stability, though specificity needs improvement (65)
Dual-Lock Integrator	Hypothesis	Whether this operates via parallel synergy or linear causality remains unresolved	Loss of both functions may explain the severity of TET-deficient phenotypes beyond TSDR remethylation alone (18, 19)	Small-molecule probes capable of distinguishing TET catalytic vs. scaffold functions represent a key tool gap for validating or falsifying this hypothesis

Evidence Level Definitions: Established = supported by multiple independent lines of evidence including *in vivo* genetic models; Emerging = supported by biochemical or correlative data, but direct *in vivo* functional validation in Tregs is incomplete; Hypothesis = inferred from indirect evidence; direct experimental validation in Tregs is pending.

Inflammatory signals, including IL-6/STAT3, may promote HDAC recruitment to Foxp3 regulatory regions and Treg-SE loci, competing with HAT-mediated active chromatin states (68). Whether this competition directly drives Treg instability requires further investigation.

### 4.2 The targeting dilemma in the tumor microenvironment

Therapeutic manipulation of TET activity presents a fundamental challenge because its biological consequences are highly context dependent (34). In autoimmune diseases, enhancing TET activity could theoretically reinforce the dual-lock architecture and promote stable regulatory function (18). In cancer immunotherapy, however, selective modulation of TET activity within tumor-infiltrating Tregs may represent a potential strategy to reduce suppressive programs (19). Importantly, systemic TET inhibition is unlikely to be feasible because TET enzymes regulate immune functions across multiple cellular compartments. TET2 exerts cell-type-specific functions across immune compartments (summarized in **Table 3**), creating a fundamental challenge for systemic targeting (62, 69).

**Table 3:** TET2 function in other tumor immunity cell types and therapeutic implications.

Cell type	Species/model	Primary function of TET2	Effect in tumor immunity	Therapeutic goal	Intervention strategy
CD8^+^ Effector T cells/TILs	Mouse (Tet2 KO)/Human	Maintains effector function and memory formation	TET2 loss enhances TIL anti-tumor activity; limits terminal exhaustion	Context-dependent (depends on differentiation stage)	Precise temporal and magnitude control; avoid systemic inhibition
NK cells	Human (MDS-associated TET2 mutations)	Maintains cytotoxicity, KIR, and perforin expression	Promotes anti-tumor innate immunity	Maintain or enhance TET2 activity	Avoid systemic TET2 inhibition
Tumor cells (certain solid tumors)	Human/Mouse	Promotes antigen presentation gene expression and IFN-γ signaling	Enhances tumor immunogenicity and T cell infiltration	Maintain TET2 activity	Avoid systemic TET2 inhibition
Myeloid-derived suppressor cells (G-MDSCs)	Mouse (Tet2 KO)	Restrains IL-6 expression; limits G-MDSC expansion	Promotes adaptive anti-tumor immunity	Maintain TET2 activity	Avoid systemic TET2 inhibition

Species abbreviations: KO, knockout; TIL, tumor-infiltrating lymphocyte; MDS, myelodysplastic syndrome; G-MDSC, granulocytic myeloid-derived suppressor cell.

**Table 3** General Note: Data summarized from Tet2 conditional knockout mouse models, human MDS patient samples, and *in vitro* human T cell experiments. The bidirectional, cell-type-dependent role of TET2 in tumor immunity necessitates compartmentalized targeting strategies rather than systemic TET2 inhibition. See main text (Section 4.2) for detailed discussion.

Addressing this challenge will require strategies capable of selectively manipulating TET activity within defined cellular populations. One potential approach involves exploiting Treg-enriched surface markers, such as CTLA-4, for targeted delivery. However, CTLA-4 is not exclusive to Tregs and can also be expressed by activated conventional T cells, limiting its specificity as a stand-alone targeting determinant. For example, CTLA-4 aptamer-conjugated STAT3 siRNA nanoparticles have been reported to reduce tumor Treg accumulation (70). More precise approaches may therefore combine CTLA-4 recognition with a second Treg-identity or tumor-associated signal, using logic-gated designs to restrict TET-modulating activity to cells exhibiting the appropriate combination of lineage and environmental features.

The CRISPRoff/CRISPRon platform has demonstrated durable and reversible epigenetic programming in primary human T cells, providing a technological foundation for future cell-selective epigenetic interventions (42, 71). However, translating these concepts into clinical applications will require careful consideration of TET’s diverse functions across immune and non-immune compartments.

## 5 Context-dependent remodeling of the dual lock in disease

The dual-lock architecture is dynamically remodeled by disease-associated signals. Inflammatory cytokines, metabolic changes, and transcriptional perturbations can alter TSDR stability and Treg-SE activity through distinct pathways. Insufficient maintenance compromises Treg fidelity in autoimmunity. Excessive reinforcement facilitates tumor immune escape. In infections, transient remodeling may reflect adaptive flexibility rather than irreversible lineage loss (**Figure 4**). Two forms of Treg dysfunction require distinction. Treg fragility refers to cells that retain Foxp3 but lose suppressive capacity. Ex-Treg conversion means irreversible Foxp3 loss with TSDR remethylation. Within our framework, fragility primarily involves Lock 2 remodeling. Ex-Treg conversion requires erosion of Lock 1. This distinction carries therapeutic implications. Fragility may be reversible. Reversing ex-Treg conversion would require epigenetic reprogramming approaches. The following sections examine how autoimmunity, tumors, and infection differentially remodel the two locks.

**Figure 4:**
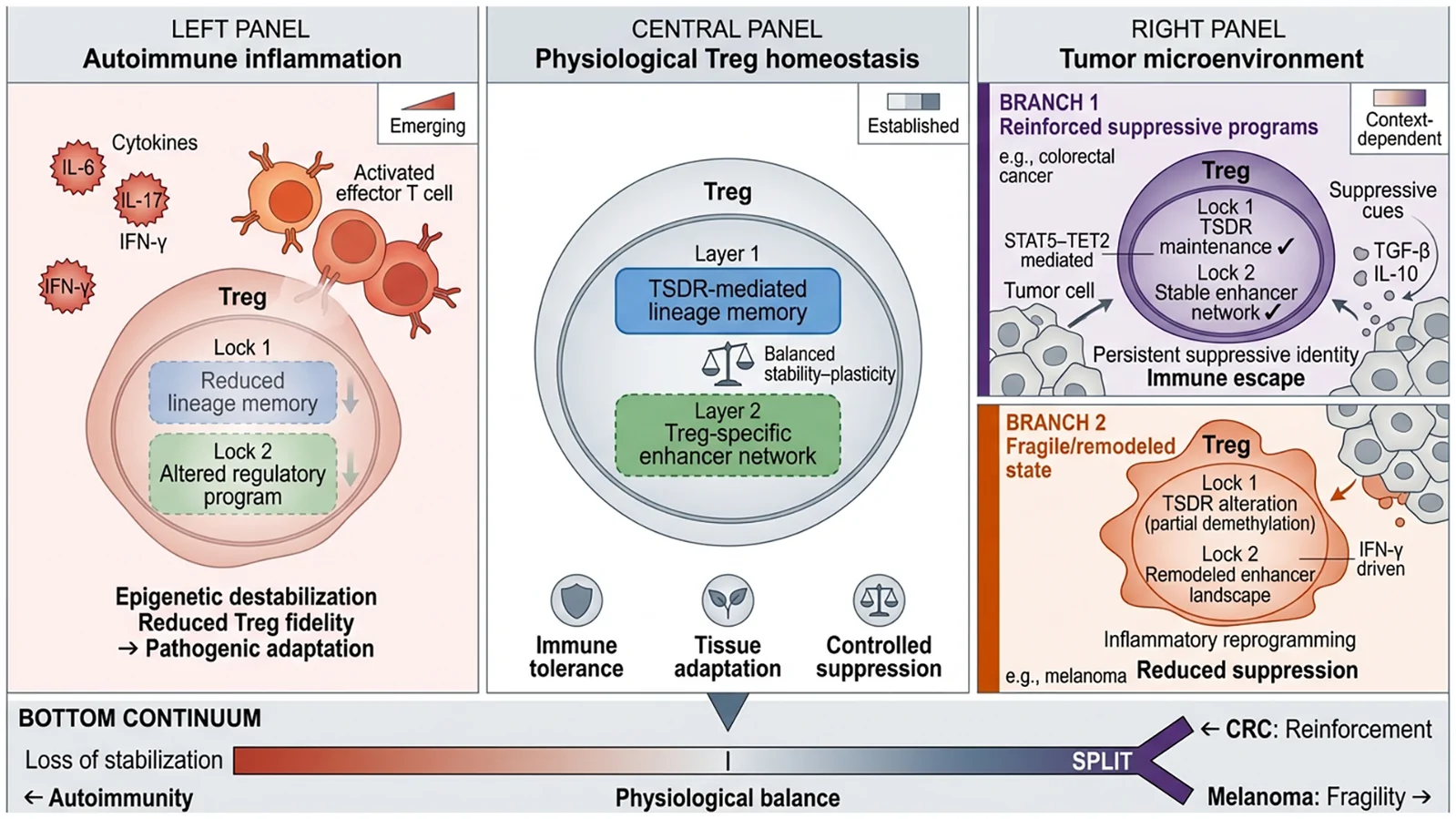
Context-dependent remodeling of the dual epigenetic lock in autoimmunity and cancer. The dual epigenetic lock architecture undergoes distinct remodeling patterns depending on disease context, illustrated across three panels. Central panel: In physiological homeostasis, balanced integration of Lock 1 (TSDR-mediated lineage memory) and Lock 2 (Treg-specific enhancer network) supports immune tolerance, tissue adaptation, and controlled suppression. Left panel: In autoimmune inflammation, inflammatory cues (IL-6, IL-17, IFN-γ) may weaken both locks, reducing Treg fidelity and potentially facilitating pathogenic adaptation. Right panel: In the tumor microenvironment, the dual lock is not uniformly reinforced. Two distinct patterns are observed: (upper branch) in tumors such as colorectal cancer, STAT5–TET2 signaling sustains TSDR maintenance and stable enhancer networks, reinforcing suppressive programs and promoting immune escape; (lower branch) in tumors such as melanoma, IFN-γ-driven signals promote Treg fragility, enhancer remodeling, and inflammatory reprogramming, leading to reduced suppression. The bottom continuum illustrates that autoimmune diseases and different tumor types occupy distinct positions along the stability–plasticity axis, emphasizing that Treg stability is not inherently beneficial or detrimental but is interpreted within its specific disease context. Evidence levels: central panel (established), left panel (emerging), right panel (context-dependent).

### 5.1 Dual lock insufficiency in autoimmune diseases

In autoimmune diseases, failure to maintain immune tolerance may partly reflect impaired integrity of the Treg dual-lock architecture. Multiple disease models and human studies suggest that defects in TSDR maintenance and broader chromatin regulation can compromise Treg stability and increase the emergence of inflammatory ex-Treg populations.

In type 1 diabetes, NOD mouse Tregs show incomplete CNS2 demethylation before insulitis starts. This suggests that epigenetic instability may appear before obvious autoimmune inflammation (72). Whether this observation fully recapitulates human T1D pathogenesis awaits confirmation in patient-derived samples. In patients with type 1 diabetes, reduced peripheral Treg TSDR demethylation is associated with decreased Foxp3 transcript levels, although the causal relationship between these alterations remains unclear (23).

Similar patterns have been observed in multiple sclerosis (MS) and experimental autoimmune encephalomyelitis (EAE). CNS-infiltrating Tregs display more pronounced epigenetic disturbances, and genetic fate-mapping studies indicate that altered DNA methylation and histone regulation contribute to Foxp3 instability and ex-Treg accumulation (19). Sumida et al. further demonstrated that abnormal DNA methylation and chromatin remodeling at the FOXP3 locus are associated with transcriptional dysfunction in Tregs from patients with MS (73). These findings support the concept that inflammatory environments can compromise both the identity-associated and functional components of the dual lock.

In rheumatoid arthritis (RA), peripheral Tregs exhibit reduced TSDR demethylation at diagnosis, accompanied by increased frequencies of Th1-like ex-Treg populations in synovial fluid. Altered methylation patterns involving TSDR and RORC regulatory regions correlate with disease activity (74–76). However, whether TSDR remethylation represents a driver of Treg dysfunction or a consequence of inflammatory remodeling remains unresolved.

Collectively, autoimmune diseases illustrate a state of dual-lock insufficiency, in which impaired maintenance of epigenetic identity and altered transcriptional regulation may reduce Treg fidelity. Importantly, available evidence does not support a universal mechanism of Treg failure; rather, the relative contribution of TSDR instability, enhancer dysregulation, and inflammatory reprogramming may vary among diseases and tissues.

### 5.2 Context-dependent remodeling of the dual lock in the tumor microenvironment

The tumor microenvironment exerts bidirectional effects on the dual-lock architecture, simultaneously generating signals that destabilize Treg programs and others that reinforce suppressive stability. This contrasts with autoimmune settings, where insufficient regulatory integrity drives pathology; in cancer, tumors frequently co-opt these very mechanisms to sustain immune evasion.

Two major inflammatory axes illustrate this duality. IL-6/STAT3 signaling induces DNMT1 expression and promotes TSDR methylation, weakening first-lock stability (68). Concurrently, IL-12-driven STAT4/T-bet activation enhances IFN-γ-associated transcriptional programs and increases Treg susceptibility to inflammatory conversion, a state termed Treg fragility (77). Conversely, tumor-associated cues can also reinforce the dual lock: IL-2/STAT5 signaling maintains TET2 occupancy at CNS2, counteracting DNMT-mediated methylation (78). In colorectal cancer (CRC), coordinated STAT5 and TET2 activity sustains TSDR hypomethylation, suggesting that tumors actively stabilize suppressive Treg programs to promote immune escape (79). Thus, the same regulatory components that preserve physiological Treg identity can be exploited by tumors, with outcome determined by signal integration within the broader microenvironment. We emphasize that the evidence linking STAT5 and TET2 to TSDR maintenance in CRC is largely correlative (79). It does not establish that tumors actively reinforce the dual lock. Alternative explanations remain plausible. The observed pattern may reflect selective outgrowth of epigenetically stable Treg clones (80). It may represent a CRC-specific cytokine milieu rather than active tumor-driven remodeling (81, 82). Distinguishing these possibilities requires longitudinal sampling and functional perturbation in autochthonous tumor models (10).

Metabolic cues within the tumor microenvironment add another layer of regulation. Severe hypoxia reduces TET activity, whereas HIF-1α activation can promote Foxp3 instability under certain conditions (46, 83). Altered glycolytic flux influences histone acetylation through acetyl-CoA availability, with ACC1 controlling the balance between lipid synthesis and chromatin modification (84). Mitochondrial metabolism further modulates epigenetic enzymes: the α-ketoglutarate-dependent activity of TET proteins is governed by mitochondrial flux, linking cellular energetics to DNA methylation dynamics (37, 85). In IDH-mutant tumors, the oncometabolite D-2-hydroxyglutarate competitively inhibits α-KG-dependent dioxygenases, suppressing TET function and potentially destabilizing Treg epigenetic states (86). These observations position metabolism as a key regulatory interface, although the direct impact of metabolic alterations on Treg-SE activity in human tumors remains incompletely defined.

The developmental composition of tumor-infiltrating Tregs may further influence how the dual lock responds to therapeutic perturbation. Tumors can contain mixtures of thymic-derived and peripherally induced Tregs (87), which may differ in their baseline TSDR configuration, antigen experience, and susceptibility to epigenetic remodeling (25). Consequently, the effect of a TSDR- or enhancer-directed intervention may depend not only on tumor type but also on the developmental composition and pre-existing epigenetic state of the Treg population (88). This possibility argues against treating all tumor-infiltrating Tregs as an epigenetically uniform compartment. However, the extent to which tTreg/pTreg composition predicts therapeutic response in human tumors remains insufficiently defined and will require lineage-aware single-cell epigenomic analyses (89).

Tumor types differ markedly in their dual-lock remodeling patterns. Melanoma features IFN-γ-driven Treg fragility and expanded ex-Treg-like populations (77), whereas CRC exhibits STAT5–TET2-associated reinforcement of suppressive programs (79). Non-small-cell lung cancer displays heterogeneous chromatin accessibility changes, including partial TSDR alteration and reduced Treg-SE activity in specific subsets, suggesting gradual remodeling rather than a binary switch (90). Importantly, parallels may exist between Treg instability and CD8^+^ T cell exhaustion, where NFAT activation without coordinated AP-1 signaling drives exhaustion programs (91). A similar imbalance could influence Treg enhancer landscapes, potentially positioning ex-Treg formation as a shared adaptive response to chronic stimulation. Nevertheless, direct evidence for this connection in human tumors remains sparse. Most mechanistic insights derive from mouse models or peripheral blood, and comprehensive epigenomic datasets from tumor-infiltrating human Tregs are limited. So, it is unclear whether the dual lock is destabilised or reinforced in human cancers. It might also be a dynamic continuum. Tumor type, stage, and treatment history could shape it. This remains an open question (**Supplementary Table S1**, Part A) (49, 77, 79, 88, 92–98).

### 5.3 Treg-to-Tfh transition: a specialized form of functional plasticity

Beyond the question of whether Foxp3 downregulation represents adaptation or failure (discussed in Section 1.1), recent evidence points to a more specific form of functional plasticity: the Treg-to-Tfh transition. In *Tet2/Tet3*-deficient Tregs, Foxp3 instability is associated with preferential differentiation toward Tfh-like states, leading to autoimmune pathology driven by autoantibody production (18, 19). This suggests that ex-Treg-derived Tfh cells may contribute to humoral immune dysregulation when lineage safeguards are compromised.

A related transition involves the acquisition of Th17-like or IL-17-producing features. In inflammatory settings, cytokine signals such as IL-6/STAT3, IL-1β, and IL-23-associated pathways may promote RORγt-associated inflammatory programs in Tregs (99). Within the dual-lock framework, such conversion can be viewed as a potential state in which inflammatory enhancer remodeling in Lock 2 precedes or accompanies erosion of Lock 1, thereby reducing the stability of Foxp3 expression (100). Importantly, this model does not imply that TSDR remethylation directly causes Treg-to-Th17 conversion. Rather, loss of TSDR-associated lineage stability and acquisition of inflammatory enhancer activity may represent partially coupled consequences of sustained inflammatory pressure (101). The observation that TET-deficient Tregs exhibit increased IL-17 expression further supports the possibility that epigenetic disruption can affect both lineage-associated and inflammatory transcriptional programs (18, 19). Whether IL-17-producing ex-Tregs represent a transient adaptive state or a stable lineage transition likely depends on the persistence and intensity of the inflammatory environment (102).

Conversely, during acute infection, a regulated Treg-to-Tfh transition may support physiological germinal center responses. Altered circulating pre-Tfh populations have been associated with anti-IFN-γ autoantibody production in severe infections (103), highlighting the context-dependent consequences of this transition. Together, these observations suggest that Treg-to-Tfh conversion may not be a passive lineage collapse. Instead, it may reflect a specialized, context-dependent adaptation. Whether it becomes pathological or beneficial depends on the regulatory milieu.

Thus, ex-Treg formation should not be viewed solely as passive lineage collapse. Shared regulatory nodes, including NFAT/AP-1 balance and TET proteins, suggest Treg state transitions are partly adaptive.Determining when such transitions represent beneficial immune flexibility versus pathological destabilization remains a central challenge for understanding Treg biology.

## 6 Technology platforms for testing the dual lock model

The dual lock model postulates two distinguishable epigenetic modules, TSDR methylation and Treg-SE chromatin conformation, that must operate in concert within the same cell. To validate the model, we need to answer three questions at single-cell resolution. First, are TSDR remethylation and Treg-SE collapse temporally coupled during ex-Treg conversion? Second, can we directly visualize Treg-SE insulation of the Ifng locus and define its breach mechanism? Third, in which tissue niches does disruption start, and what signals drive it? An integrated experimental strategy addressing these questions, spanning single-cell multi-omics, spatial epigenomics, and functional perturbation, is outlined in **Figure 5**.

**Figure 5:**
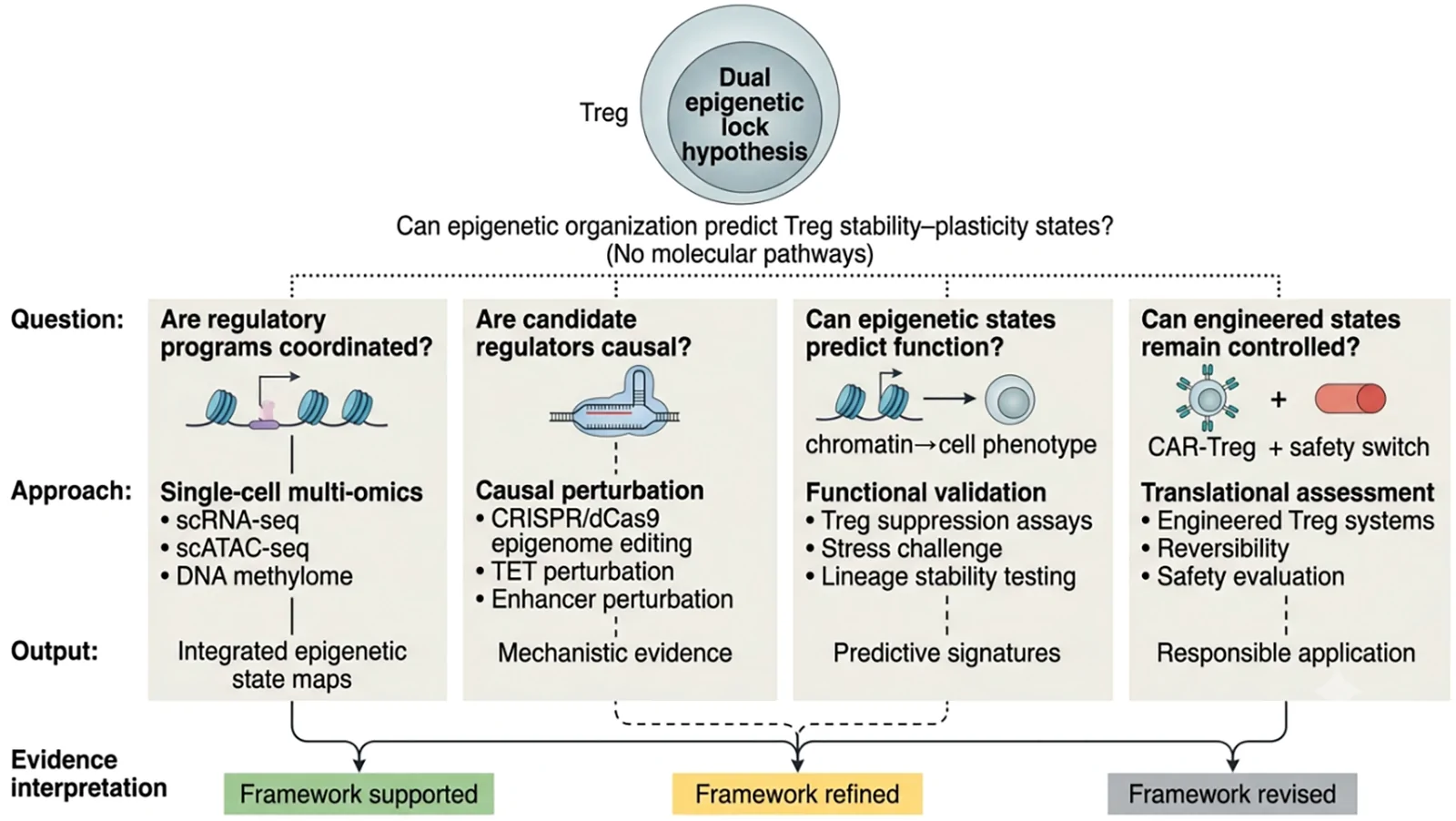
Experimental roadmap for testing and refining the dual epigenetic lock hypothesis. The dual epigenetic lock hypothesis generates testable questions about regulatory coordination, causality, predictive capacity, and translational feasibility. Integrative multi-omics can define epigenetic states. Perturbation strategies can establish causality. Functional assays and engineered Treg systems can evaluate whether epigenetic configurations predict cellular behavior and therapeutic controllability. A critical component of this roadmap is defining falsification criteria. If TSDR and Treg-SE states can be manipulated independently without cross-regulation, Model C would be falsified in favor of Model (B) If TSDR manipulation alone fails to reconstitute full Treg function while combined modulation succeeds, the integrated architecture would be supported. These studies will determine whether the framework is supported, refined, or revised. Developmental origin, metabolic state, and tissue-specific environmental signals should be incorporated as upstream variables when testing whether dual-lock configurations predict Treg-state transitions.

### 6.1 Capturing epigenetic conversion trajectories

Single-cell transcriptomics show that ex-Tregs are not binary. They exist along a continuum of transitional states. Foxp3 programs gradually decline while inflammatory features progressively appear (44). These findings suggest that remodeling of the dual lock may occur progressively rather than through an abrupt transition. Computational approaches such as RNA velocity can further infer the directionality and dynamics of Treg-state transitions, providing insight into potential trajectories of Treg-to-ex-Treg conversion (104).

However, transcriptional trajectories alone cannot determine the underlying epigenetic states of the dual lock. Similar transcriptional phenotypes may arise from distinct regulatory mechanisms, including altered DNA methylation, enhancer remodeling, or transient transcriptional adaptation (5, 105).

To investigate the relationship between transcriptional states and epigenetic configuration, single-cell multi-omics approaches such as scMultiome enable simultaneous measurement of transcriptomes and chromatin accessibility within the same cell (21). These approaches provide a framework for assessing whether transcriptional changes associated with ex-Treg transition coincide with alterations in regulatory chromatin landscapes.

Complementary DNA methylation-based approaches are required to directly examine TSDR status. Single-cell bisulfite sequencing (scBS-seq) can determine whether phenotypically converted ex-Tregs undergo TSDR remethylation and whether such changes precede or follow Foxp3 downregulation (106, 107). Resolving this temporal relationship requires lineage tracing (30). Sparse scBS-seq coverage can be addressed by deeper sequencing. A more fundamental challenge is ordering events in time. A cell may lose Foxp3 before TSDR remethylation, or remethylation may precede Foxp3 loss (108). Combining scBS-seq with a division reporter would distinguish these scenarios. This would reveal whether TSDR remethylation drives instability or merely accompanies it.

### 6.2 Resolving the molecular architecture of the dual lock

Beyond identifying epigenetic states, understanding the dual-lock model requires direct assessment of the regulatory architecture that connects transcription factors, enhancers, and target genes.

Foxp3 HiChIP has provided evidence that Foxp3 participates in enhancer–promoter interactions at key Treg-associated loci, including *Foxp3*, *Ctla4*, and *Il2ra* (16). These findings support a role for Foxp3 in shaping higher-order chromatin organization (16), although the extent to which these interactions determine Treg stability remains an active area of investigation.

Chromatin profiling technologies further enable characterization of enhancer-associated regulatory states. CUT&Tag maps histone marks like H3K27ac at Treg-SEs with high resolution. It needs fewer cells than ChIP-seq. It also gives better signal-to-noise ratios (109). Combined with chromatin accessibility profiling, these approaches can reveal how Treg-SE landscapes are altered during activation, adaptation, or inflammatory conversion.

Together, chromatin conformation and epigenomic profiling technologies provide complementary strategies for determining how the structural integrity of Treg-SE networks changes during Treg-state transitions. Importantly, these approaches may distinguish whether functional instability arises from loss of enhancer activity, altered chromatin organization, or disruption of specific regulatory interactions.

### 6.3 Mapping dual-lock remodeling in tissue contexts

The dual-lock model predicts that Treg stability is shaped not only by intrinsic regulatory programs but also by spatially restricted microenvironmental signals. Therefore, defining where dual-lock remodeling occurs within tissues is essential for understanding its biological consequences.

Multiplexed imaging technologies, including CODEX and MIBI, have enabled spatial characterization of immune cell states within complex tissue ecosystems (110). Studies of tumor tissues have revealed spatial enrichment of Treg populations with altered Foxp3 expression patterns at specific anatomical niches, including tumor–immune interfaces and invasive margins. These observations suggest that local environmental conditions may influence Treg-state transitions.

Emerging spatial epigenomic technologies extend this analysis by enabling molecular profiling within intact tissue architecture. Spatial CUT&Tag allows mapping of histone modifications in situ (111), whereas spatial ATAC-RNA-seq integrates chromatin accessibility with transcriptional information at cellular resolution (112). These approaches provide the potential to directly associate dual-lock states with specific tissue niches, cellular interactions, and environmental signals.

Future studies must integrate spatial, temporal, and molecular measurements. Finding where the dual lock remodels is just the first step. We also need to know when it starts and what signals drive it. That will help us tell adaptive plasticity from pathological instability.

A decision framework linking experimental objectives with appropriate technological approaches is summarized in **Table 4**.

**Table 4:** Core technologies for validating the dual lock model—summary and decision tree.

Technology	Detection content	Model aspect validated	Resolution	Throughput	Key advantages	Major limitations	Recommended application
scRNA-seq	Transcriptome	Cell state classification (ex-Treg identification)	Single-cell	High	Simultaneous detection of thousands of genes; mature analytical ecosystem	No epigenetic information; cells with low Foxp3 expression may be filtered as background	Initial identification of ex-Treg populations; mapping the conversion continuum
CITE-seq	Transcriptome + surface proteins	ex-Treg-specific surface markers	Single-cell	High	Joint transcriptome and protein analysis; enables sorting of ex-Tregs via unique markers	Antibody panel design determines detection scope; poor antibody quality affects signal-to-noise	Discovery and validation of ex-Treg surface markers
scATAC-seq	Chromatin accessibility	Second lock (SE activity)	Single-cell	Medium	Directly detects openness dynamics at Treg-SEs and the *Ifng* locus	No DNA methylation information; complex data processing	Characterizing Treg-SE activity changes; discovering heterogeneous subpopulations
scBS-seq/scWGBS	DNA methylation	First lock (TSDR demethylation)	Single-base	Low	Directly detects methylation status of individual CpGs	High cost; limited coverage; only detects methylation	Verifying the causal link between TSDR remethylation and Foxp3 loss
Single-cell multi-omics (scMultiome)	Transcriptome + chromatin accessibility (same cell)	Correlation between the two locks	Single-cell	Low-Medium	Correlates expression and epigenetic state in the same cell	Data sparsity; technology is rapidly iterating	Associating epigenetic changes with transcriptional shifts during ex-Treg conversion
HiChIP/Hi-C	3D chromatin conformation	Enhancer-promoter looping of the second lock	Population/single-cell	Low	Direct evidence: intactness of enhancer-promoter loops	Large cell input requirement; complex analysis; currently mainly population-based	Validating spatial contacts or insulation between Treg-SEs and target genes (e.g., *Foxp3*, *Ifng*)
CUT&Tag	Histone modifications (e.g., H3K27ac)	Activity status of the second lock	Population/single-cell	High	Low cell input; superior signal-to-noise ratio vs. ChIP-seq; selective for H3K27ac	Dependent on high-quality antibodies; single-cell throughput currently relatively low	Rapid validation of Treg-SE activity status
CODEX/MIBI	Spatial co-expression of 40–100 proteins	Spatial distribution of ex-Tregs	Single-cell (spatial preservation)	Medium	Spatial resolution (*in situ*); protein-level detection	Antibody panels require pre-experimental validation; complex data analysis	*In situ* identification of ex-Tregs and their spatial relationship with the microenvironment
Spatial transcriptomics (10x Visium, MERFISH)	Spatially resolved transcriptome	Spatial enrichment zones of ex-Tregs	55 μm (Visium) to single-cell (MERFISH)	High	Unbiased discovery of ex-Treg spatial distribution; reveals spatial niches driving conversion	Currently mainly provides transcriptional information; lacks direct epigenetic evidence	Discovering tissue microenvironments that drive dual lock disruption

Memory and tissue-resident Tregs provide an additional context in which stability and plasticity must be considered together. These populations can maintain durable lineage-associated features while undergoing substantial transcriptional and functional adaptation to their local tissue environment (22). Within the dual-lock framework, Lock 1 can therefore be viewed as contributing to lineage memory by preserving Foxp3 expression competence (28), whereas Lock 2 provides a more flexible regulatory layer through which tissue-specific signals shape suppressive and effector functions. This interpretation does not equate TSDR demethylation with tissue residency; rather, it predicts that stable lineage-associated chromatin can coexist with substantial remodeling of tissue-specific enhancer programs (113). Determining how these two layers are maintained during repeated antigen exposure and tissue-specific recall responses will be an important test of the framework (114).

## 7 Therapeutic strategy I: “reinforcing the lock” in autoimmune diseases

If impaired Treg stability contributes to the breakdown of immune tolerance, a logical therapeutic strategy is to convert this biological vulnerability into a therapeutic opportunity by reinforcing regulatory stability. The objective, however, is not to generate an irreversibly fixed regulatory cell, but rather to create Tregs with controllable stability—cells that preserve suppressive identity while remaining responsive to physiological cues.

CAR-Tregs have shown early evidence of feasibility and safety in clinical studies for autoimmune diseases and transplantation settings (115, 116). However, adoptively transferred Tregs may gradually lose Foxp3 expression or acquire ex-Treg features, posing a major challenge for durable therapeutic efficacy (5, 6). This loss can arise from distinct mechanisms: tonic or antigen-specific CAR signaling may drive activation-induced exhaustion and functional attenuation, whereas the inflammatory microenvironment (e.g., IL-6/STAT3 signaling) imposes a sustained stress that directly challenges lineage fidelity. Distinguishing these pathways is essential for designing CAR-Treg products with optimized persistence (99, 117). Therefore, lock-reinforcing strategies should aim not simply to maximize stability, but to establish a balanced regulatory state that combines lineage fidelity, environmental responsiveness, and controllability.Three principles guide these approaches. First, avoid irreversible fixation. Second, keep responsiveness to tissue signals. Third, build in safety switches for selective control or removal when needed.

### 7.1 Metabolic reinforcement

Metabolic interventions can enhance epigenetic stability by modulating the enzymatic environment required for Treg identity maintenance. The ‘3C’ strategy combines hypoxia, TET catalytic domain overexpression, and vitamin C. It drives extensive CNS2 demethylation in induced Tregs. This improves Foxp3 stability and suppressive function after inflammation (14, 35, 36) (see Section 2.2 for TET mechanism). Such approaches could be incorporated into ex vivo CAR-Treg manufacturing workflows to enhance therapeutic Treg stability before infusion. However, whether engineered epigenetic reinforcement persists after transfer into complex inflammatory environments remains an open question.

Beyond DNA methylation regulation, metabolic programming itself influences Treg persistence and function (118). One potential strategy is to modulate glycolysis versus OXPHOS, including mildly inhibiting glycolysis or boosting CPT1a-dependent fatty acid oxidation (FAO). FAO may influence the availability of mitochondrial metabolites and cofactors that support epigenetic enzyme activity (119), whereas fatty acid synthesis (FAS) can alter acetyl-CoA availability and thereby affect histone acetylation and enhancer activity (84). These pathways could therefore influence both locks indirectly by modifying the biochemical environment for TET-dependent DNA demethylation and histone-based regulation of Treg-associated enhancers (119, 120). However, direct evidence that FAO or FAS coordinately controls TSDR status and Treg-SE activity in primary Tregs remains limited. Metabolic reinforcement should maintain metabolic flexibility rather than drive cells toward a single metabolic state (118).

### 7.2 Genetic engineering

Targeted epigenetic editing provides a more direct approach to manipulate components of the dual-lock architecture. CRISPR-dCas9-TET1 fusion systems enable targeted modulation of TSDR methylation states, offering a strategy to reinforce the first lock without globally altering DNA methylation.

Arroyo-Olarte et al. used this method in STAT6-deficient iTregs. They found that targeted FOXP3-TSDR demethylation boosted Treg-related genes. These included Foxp3, CTLA-4, PD-1, IL-10, and TGF-β (43). However, dCas9-TET1 catalytic domain targeting alone produced incomplete stabilization, suggesting that DNA demethylation may be insufficient to fully reconstruct the broader Treg regulatory program (42).

Consistent with the dual-lock framework, simultaneous reinforcement of DNA methylation status and enhancer activity may provide greater stability than targeting either layer independently. For example, combining dCas9-based TET activity with enhancer-modulating strategies such as p300-mediated histone acetylation could theoretically strengthen both lineage-associated and functional regulatory modules. However, several limitations must be acknowledged before clinical translation. First, off-target demethylation at genomically similar loci remains a concern, as dCas9-TET1 fusions can exhibit residual activity at non-target sites (121). Second, the durability of episomally expressed dCas9 systems may be transient, potentially requiring repeated administration or integration into safe-harbor loci for sustained effect (122). Third, the stoichiometry of TET catalytic domain versus full-length TET2/TET3—which may possess non-catalytic scaffolding functions—remains unexplored in the Treg context (123). Addressing these issues will require systematic off-target profiling, optimized guide RNA design, and perhaps next-generation epigenome editors with enhanced specificity.

Synthetic receptor engineering represents another approach aligned with the principle of maintaining environmental responsiveness. OX40L-specific CAR-Tregs driven by a synthetic FOXP3 promoter have been reported to maintain suppressive activity under inflammatory conditions by integrating external activation signals with reinforcement of Treg-associated programs (124). Such designs illustrate a broader concept: rather than isolating Tregs from inflammatory environments, engineered circuits may enable Tregs to interpret environmental signals while preserving regulatory function.

### 7.3 Synthetic biology and safety

A future direction for lock-reinforcing strategies is the development of programmable synthetic circuits that integrate multiple layers of regulatory control. A conceptual design uses genome-encoded stability modules. These include TET2 catalytic domain, p300 HAT domain, and dominant-negative STAT3. They are placed at defined loci like the AAVS1 safe-harbor site (**Figure 6**) (125, 126).

**Figure 6:**
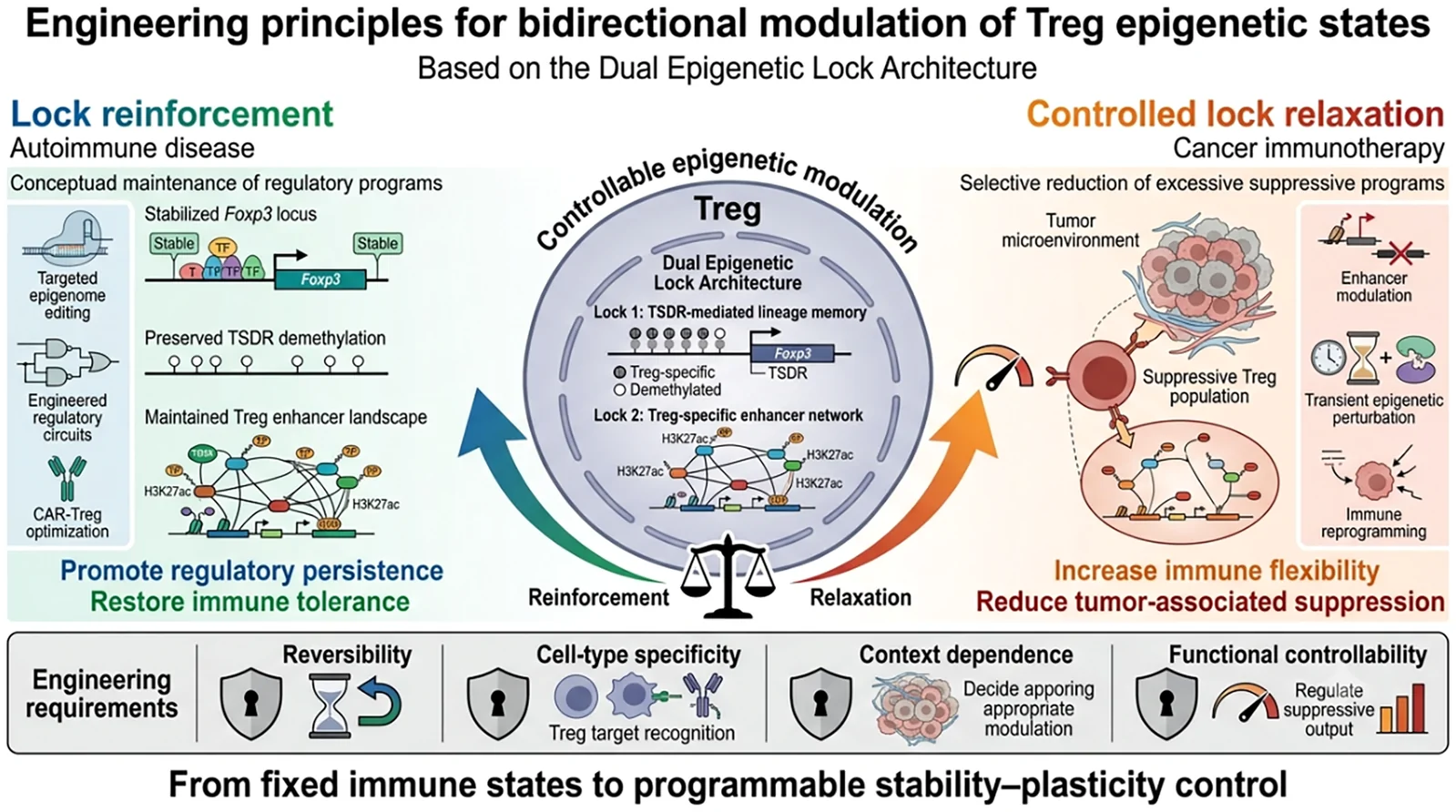
Engineering principles for bidirectional modulation of Treg epigenetic states. Lock-reinforcing is not a single technique, but rather a progressively layered toolkit spanning from metabolism to synthetic biology. Centered around a CAR-Treg cell, metabolic reinforcement involves enhancing TET activity with vitamin C, modulating the OXPHOS/glycolysis balance to maintain a metabolically quiescent phenotype, and promoting fatty acid oxidation to increase persistence—strengthening the dual lock at the level of biochemical substrates and enzymatic activity. Genetic reinforcement involves CRISPR-dCas9-TET1 targeted demethylation, p300/CBP overexpression to enhance H3K27ac, STAT5-responsive Foxp3 expression systems, and chimeric cytokine receptors that convert IL-6/STAT3 inhibitory signals into STAT5 activating signals—directly fortifying the molecular architecture of both locks. Synthetic biology reinforcement involves integrating a synthetic enhancer circuit driven by Treg-specific transcription factors at the AAVS1 safe-harbor locus to express the TET2 core, p300 core, and dnSTAT3, equipped with a built-in suicide switch to ensure controllability. Three guiding design principles run throughout: avoid irreversible fixation of Treg identity; maintain environmental responsiveness to local cues; and include built-in safety switches enabling selective elimination. The core design philosophy is to create a cell that is stable yet controllable, with safety as the starting point.

However, increasing the strength of stability engineering also increases the importance of defining safety boundaries. Excessively reinforced Treg suppressive activity could potentially impair immune surveillance and compromise host defence mechanisms (118, 127). Therefore, therapeutic stability must be coupled with mechanisms that ensure controllability.

Preclinical studies highlight both the promise and limitations of engineered Tregs. In a humanized systemic lupus erythematosus model, CD19-targeted CAR-Tregs reduced autoantibody production, although limited *in vivo* persistence restricted therapeutic durability (128). Similarly, HLA-A2 CAR-Tregs engineered with a chemically inducible IL-2 signaling module achieved antigen-specific suppression while minimizing bystander immune effects (129). These studies demonstrate that enhancing specificity and persistence remains a central challenge for clinical translation.

Next-generation armored Tregs may require multiple layers of safety control. The inducible caspase-9 (iCasp9) system provides an established strategy for conditional elimination of engineered cells (130, 131). Future designs may incorporate additional safeguards, including metabolic dependency switches, logic-gated apoptotic circuits, antigen-dependent survival modules, or surface markers such as truncated EGFR or CD20 that enable antibody-mediated clearance.

Platforms like CRISPRoff/CRISPRon enable durable but reversible gene regulation in human T cells. They offer a technological basis to dynamically control engineered Treg states (42, 71). Ultimately, reinforcing the lock needs a balance of stability and flexibility. We do not aim for permanently fixed suppressive cells. Instead, we want engineered Tregs that are stable, adaptable, and controllable.

## 8 Therapeutic strategy II: “precision unlocking” in cancer immunotherapy

Unlike lock reinforcement, precision unlocking aims to selectively disrupt tumor-associated Treg suppressive programs while preserving systemic immune regulationtumor (132–134). The goal is a calibrated reduction of Treg-mediated immunosuppression, sufficient to restore anti-tumor immunity without triggering uncontrolled autoimmunity (135, 136). Two mechanistically distinct approaches fall under this umbrella. Treg depletion physically removes tumor-infiltrating Tregs. Epigenetic unlocking modifies the regulatory state of existing Tregs. The dual-lock framework primarily informs the latter, predicting which epigenetic nodes destabilize suppressive programs. It also provides a rationale for combining depletion with epigenetic modulation. Two complementary strategies emerge from this framework: surface marker-directed depletion and functional epigenetic reprogramming. The spectrum of current approaches is summarized in **Figure 7**.

**Figure 7:**
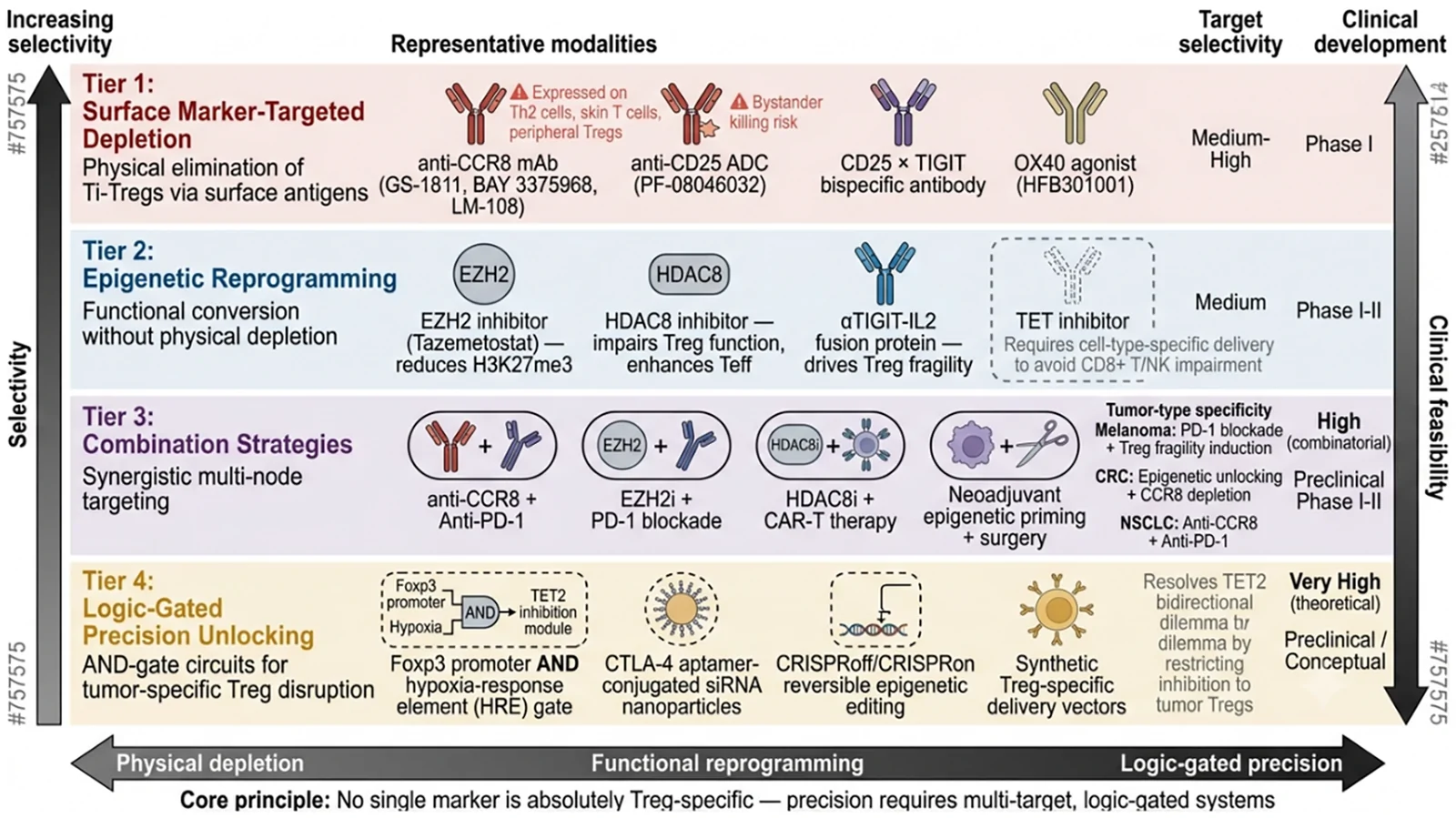
Therapeutic strategy spectrum for precision unlocking in cancer immunotherapy. The spectrum spans four tiers of increasing selectivity: surface marker-targeted depletion (Tier 1, Phase I), epigenetic reprogramming (Tier 2, Phase I–II), combination strategies (Tier 3, preclinical to Phase I–II), and logic-gated precision unlocking (Tier 4, preclinical/conceptual). Tumor-type-specific strategy adaptations are indicated for melanoma, colorectal cancer (CRC), and non-small cell lung cancer (NSCLC). The core principle is that no single surface marker is absolutely Treg-specific, necessitating multi-target, logic-gated systems for true precision. Established clinical modalities are shown with solid outlines; conceptual approaches with dashed outlines.

### 8.1 Surface marker-targeted depletion

Surface-marker-directed depletion represents the unlocking strategy currently closest to clinical translation. CCR8 is the most clinically advanced target, with preferential (though not exclusive) expression on tumor-infiltrating Tregs. Kidani et al. showed that tumor-reactive Tregs express high levels of CCR8. Anti-CCR8 antibodies selectively reduce these cells. They spare most effector T cells and non-tumor Tregs. This led to better tumor control and tumor-reactive immunity in preclinical models (132).

Translational studies further support this strategy. Campbell et al. showed that Fc-optimized, non-fucosylated anti-human CCR8 antibodies can selectively deplete CCR8-expressing Tregs in ex vivo tumor cultures derived from human specimens (133). Several anti-CCR8 antibodies are now in early clinical trials. These include GS-1811, BAY 3375968, and LM-108 with anti-PD-1. Preliminary anti-tumor activity has been reported in some tumor types (134, 135).

However, CCR8 is not completely restricted to Ti-Tregs. It may also be expressed by subsets of Th2 cells, tissue-resident T cells, dysfunctional intratumoral CD4^+^ and CD8^+^ T cells, and peripheral Treg populations (137, 138). Therefore, long-term assessment of immune perturbations and tissue-specific effects will be essential as CCR8-directed therapies progress clinically. A comparative risk assessment of CD25, CCR8, and other targets is presented in **Supplementary Table S1**, Part B (132, 139–149). It is noteworthy that CCR8 is also expressed on subsets of Th2 cells and tissue-resident memory T cells in the skin, raising potential on-target/off-tumor toxicity risks such as dermatitis (139). Early-phase clinical trials of anti-CCR8 agents should therefore incorporate vigilant monitoring of skin and mucosal tissues, including serial dermatologic examinations and patient-reported pruritus and rash assessments. Whether such toxicity can be mitigated by Fc-optimized antibodies that require higher CCR8 density for effector function, or by combination with localized rather than systemic administration, remains to be determined (150).

Targeting CD25 presents additional challenges. Although CD25 is highly expressed on activated Tregs, activated effector T cells can also upregulate this receptor, limiting selectivity. PF-08046032 is an affinity-modulated anti-CD25 ADC. It aims to preferentially target CD25-high tumor Tregs. But bystander toxicity from payload release is still a major safety concern (140).

Bispecific antibody approaches may improve selectivity by integrating multiple recognition signals. For example, CD25×TIGIT bispecific antibodies preferentially target CD25^+^TIGIT^+^ regulatory populations and have shown enhanced anti-tumor activity with reduced effects on peripheral Tregs in preclinical studies (136). These strategies illustrate a broader principle of precision unlocking. To discriminate tumors selectively, we will likely need combinatorial recognition. Relying on a single marker will not be enough.

### 8.2 Epigenetic unlocking

Whereas surface-targeting approaches primarily reduce the abundance of suppressive Tregs, epigenetic strategies aim to modify the regulatory state that maintains Treg suppressive function. Conceptually, this approach could redirect tumor-associated Tregs toward a less suppressive phenotype while preserving aspects of immune regulation.

Targeting TET activity represents one potential route to weaken the first lock by promoting TSDR remethylation. However, systemic TET inhibition remains problematic because TET proteins also support anti-tumor functions in other immune compartments, including CD8^+^ T cells and NK cells (19, 62, 63). Therefore, therapeutic application will require cell-type-selective approaches capable of distinguishing tumor-associated Tregs from beneficial immune populations.

Histone-modifying enzymes provide additional opportunities for epigenetic intervention. HDAC8 inhibition has been reported to reduce Treg suppressive activity while enhancing effector T-cell responses, suggesting that modulation of chromatin accessibility may influence both regulatory and effector compartments (65, 151). Similarly, an αTIGIT–IL-2 fusion protein has been shown in preclinical models to promote tumor control partly through induction of Treg fragility and functional destabilization (152).

EZH2 represents another chromatin regulator involved in Treg functional maintenance. EZH2 inhibition, including approaches involving tazemetostat-sensitive pathways, can remodel Treg-associated chromatin states by reducing H3K27me3 deposition. This concept has demonstrated preclinical activity and is being explored in clinical studies (NCT05934838, NCT05994235) (153, 154).

Nevertheless, epigenetic unlocking requires careful consideration of context. Because chromatin regulators frequently control multiple immune lineages, broad inhibition may produce unpredictable effects. The future of epigenetic unlocking will likely depend on targeted delivery systems, transient modulation, or programmable approaches that restrict intervention to tumor-associated regulatory populations.

### 8.3 Combination strategies and tumor-type specificity

Because tumor immune evasion involves multiple regulatory layers, single-agent unlocking strategies are unlikely to achieve optimal therapeutic effects. Combining selective Treg targeting with immune checkpoint blockade represents one of the most actively investigated approaches.

CCR8-directed depletion combined with PD-1 blockade currently represents one of the most extensively studied combinations, supported by preclinical evidence and emerging clinical evaluation (135, 155). By reducing tumor-associated suppressive populations while simultaneously restoring exhausted effector T-cell activity, this strategy aims to address complementary barriers within the tumor immune microenvironment.

Epigenetic modulators may provide additional synergy. Combining EZH2 or HDAC inhibitors with CAR-T-cell approaches or PD-1 blockade could potentially enhance anti-tumor immunity by altering suppressive regulatory programs while improving effector-cell function (65, 151, 154). However, the optimal sequence and intensity of these interventions remain to be determined.

Importantly, unlocking strategies will likely require adaptation to tumor-specific patterns of dual-lock remodeling. Different tumors exploit distinct regulatory mechanisms. Melanoma shows increased Treg fragility and inflammatory reprogramming, so it may respond to Treg-destabilizing approaches (77). Colorectal cancer may rely more on epigenetic reinforcement, supporting combinations of epigenetic modulation and CCR8 depletion (79, 156). Non-small-cell lung cancer may benefit from anti-CCR8 plus PD-1 blockade (155).

A fundamental limitation remains the lack of a single marker capable of completely distinguishing tumor-associated Tregs from peripheral Tregs or activated effector T cells. Thus, future precision unlocking will likely need multi-parameter recognition. This means combining surface markers, transcriptional states, and tissue signals. This is the logic-gated tier in our strategy spectrum (**Figure 7**, Tier 4). Addressing this specificity challenge will be essential for translating precision unlocking from a conceptual framework into clinically effective cancer immunotherapy.

## 9 Future directions and concluding remarks

### 9.1 Priority research directions

Beyond resolving the architectural relationship, five testable hypotheses are prioritized. (i) Developing dual-fluorescence reporter mice that simultaneously track TSDR methylation status and Treg-SE activity, enabling longitudinal monitoring of whether first-lock disruption precedes or follows second-lock collapse during inflammatory challenge. (ii) Use Foxp3 fate-mapping to see if ex-Treg-derived Tfh cells help germinal center responses. This tests whether Treg plasticity is a regulated adaptation, not a pathological failure (18, 103). (iii) Engineering logic-gated AND-gate circuits (Foxp3 promoter + hypoxia-response element) for tumor-specific epigenetic unlocking, addressing the fundamental challenge of cell-type selectivity without systemic toxicity (124, 157). (iv) Compare lymphoid and non-lymphoid tissues using single-cell multi-omics to determine whether tissue-resident Tregs maintain distinct enhancer configurations while preserving lineage-associated TSDR states (158). This will test whether tissue adaptation can be accommodated within the two-lock architecture or requires additional regulatory layers (22). (v) Embed mechanistic correlative studies in ongoing trials. Collect tissue biopsies systematically for single-cell multi-omics. This will fill the gap in human dual-lock data from diseased tissues.

### 9.2 Concluding remarks

The dual-lock model places immune homeostasis within a tunable epigenetic architecture: TSDR demethylation provides a static lineage imprint, while the super-enhancer network enables dynamic functional regulation. This architecture, shaped continuously by microenvironmental and metabolic signals, is not a fixed circuit. From this logic emerge two symmetric therapeutic strategies: lock reinforcement in autoimmunity, precision unlocking in cancer. Both approaches demand controllable modulation of epigenetic states—engineering stability without rigidity, and disruption without systemic toxicity. As single-cell spatial multi-omics, epigenome-editing tools, and next-generation animal models mature, the dual-lock framework will evolve into a quantitatively testable entity. The model’s enduring value may lie in this iterative refinement from concept to validated paradigm.
