# Supplementary material for: Beyond Foxp3 and TSDR: a dual epigenetic lock framework for treg stability and plasticity
Source: Front Immunol. 2026 Sep 22;17:1952451. doi: 10.3389/fimmu.2026.1952451 (PMC13640018; doi:10.3389/fimmu.2026.1952451)
Supplement: Supplementary file 1 [file Table1.docx]

**Supplementary Table S1: Evidence Gap Analysis of Treg Dual Lock Disruption in the Human Tumor Microenvironment and Target Safety Assessment for Precision Unlocking**

**Part A: Evidence Cross-Comparison and Gap Analysis of Treg Dual Lock Disruption in the Human Tumor Microenvironment**

*(Cross-reference: Main text Section 5.2, "Context-Dependent Remodeling of the Dual Lock in the Tumor Microenvironment" and "A Critical Gap to Bridge" paragraph)*

| Evidence Category | Evidence Supporting Lock Disruption | Species / Sample Source | Evidence Supporting Lock Maintenance or Hijacking | Species / Sample Source | Evidence Strength | Critical Methodological Gaps | Priority Future Directions |
| --- | --- | --- | --- | --- | --- | --- | --- |
| First Lock: TSDR Methylation Status | • Eight CpG sites within the Foxp3 promoter demethylated in peripheral blood CD4⁺ Tregs from NSCLC patients, with methylation ratios negatively correlated with Treg proportions(143)  • Increased heterogeneity of Foxp3 methylation detected in Tregs from certain tumor types (88) | Human (peripheral blood) / Human (tumor types unspecified) | • In CRC patients, STAT5 and TET2 are synergistically overexpressed in tumor-infiltrating CD4⁺ T cells and cooperatively bind to FOXP3-TSDR, leading to TSDR-specific DNA hypomethylation that stabilizes Foxp3 expression (79)  • Multiple studies indicate that tumor Tregs globally maintain a hypomethylated TSDR status | Human (tumor-infiltrating, CRC) / Review | Moderate-to-weak: Majority of direct evidence from mouse models; WGBS data from human tumor Tregs are exceedingly scarce; NSCLC evidence from peripheral blood, not intratumoral | • Purified human tumor Treg genome-wide methylation data (scWGBS) urgently needed  • No published HiChIP/Hi-C or CUT&Tag data from human tumor Tregs  • Simultaneous detection of TSDR methylation and Foxp3 expression in the same single cell required to establish a causal chain | ① Purified human tumor Treg scWGBS across multiple cancer types  ②Simultaneous scMultiome (ATAC + RNA + methylation) in tumor Tregs  ③HiChIP/CUT&Tag studies on human tumor Tregs to verify enhancer-promoter looping status |
| Second Lock: Treg-SE Activity (H3K27ac) | • Comparative analyses between tumor Tregs and normal tissue Tregs reveal H3K27ac mark redistribution at enhancer regions (49)  • In vitro pro-inflammatory cytokine treatment can induce H3K27ac remodeling at human Treg SE regions | Human (tumor-infiltrating + in vitro) / In vitro | • Integrative epigenomic profiling of human CRC and NSCLC tumor Tregs indicates that tumor Treg-specific active enhancers remain open in tumor tissues (49,144)  • Although the H3K27ac landscape of tumor-infiltrating Tregs differs significantly from peripheral Tregs, the H3K27ac signal at core Treg-SE regions is not lost in tumor Tregs | Human (tumor-infiltrating, CRC, NSCLC) | Weak: Direct histone modification and three-dimensional genomic data from human tumor Tregs severely lacking | • No HiChIP or Hi-C data from human tumor Tregs available • CUT&Tag for H3K27ac not performed on purified human tumor Tregs  • Spatial transcriptomics integration with SE activity validation needed | ①HiChIP/CUT&Tag studies on human tumor Tregs to directly verify enhancer-promoter looping status  ② Integration with spatial transcriptomics to validate SE activity within tumor tissues |
| Dual Lock Synergy Marker: TET Enzyme Expression and Function | • Hypoxia and D-2-HG can inhibit human T cell TET activity in vitro (145)  • Reduced TET2 expression in certain tumor microenvironments correlates with diminished Treg stability(146) | Human (in vitro) / Human (tumor-infiltrating) | • STAT5 and TET2 are synergistically overexpressed in CRC to maintain TSDR hypomethylation (79)  • TET2 maintains high expression in Tregs across multiple solid tumor types to consolidate the immunosuppressive phenotype | Human (tumor-infiltrating, CRC) / Review | Moderate-to-weak: TET activity dynamically regulated by the microenvironment; in vitro data difficult to extrapolate directly to in vivo; methods for direct detection of intratumoral TET activity in humans absent | • Probes or methods capable of directly detecting TET catalytic activity (5hmC/5fC/5caC) in vivo needed  • No methods to distinguish TET catalytic vs. scaffold function in live human Tregs | ① Pan-cancer single-cell multi-omics to systematically compare TET expression and activity across tumor types  ②Development of activity-based TET probes for in vivo detection |
| ex-Treg Conversion and Functional Status | • Co-expression of T-bet and IFN-γ elevated in Foxp3⁺ T cells from human melanoma and peripheral blood(77)  • Microbiota-specific antigens drive Treg→Tfh-like cell conversion with Foxp3 loss in human CRC(147)  • scRNA-seq reveals a transcriptional continuum from Foxp3-high to effector-like states in human tumor Tregs (148) | Human (tumor-infiltrating, melanoma) / Human (tumor-infiltrating, CRC) / Human (tumor-infiltrating, melanoma) | • The majority of human tumor Tregs maintain stable Foxp3 expression, with ex-Tregs constituting only a small fraction  • Co-elevation of FOXP3 and BATF in melanoma leads to activation of Treg self-maintenance genes and immunosuppressive molecule expression (149) | Review / Human (tumor-infiltrating, melanoma) | Moderate: scRNA-seq provides evidence of transcriptional state heterogeneity, but direct epigenetic validation insufficient | • Simultaneous capture of transcriptome and epigenome in the same cell needed to verify whether ex-Treg conversion is accompanied by dual lock disruption  • Spatial multi-omics required to correlate spatial localization of ex-Tregs with their microenvironmental signals | ①Simultaneous scMultiome in tumor Tregs to verify epigenetic basis of ex-Treg conversion  ② Spatial multi-omics to correlate ex-Treg localization with microenvironmental signals |
| Cross-Tumor-Type Comparison | • IFN-γ-driven Treg fragility more pronounced in melanoma (77)  • Tregs partially retain suppressive phenotype in NSCLC | Human (tumor-infiltrating, melanoma) / Human (tumor-infiltrating, NSCLC) | • Tumor Tregs in CRC exhibit a highly stable TSDR hypomethylation status (79)  • Epigenetic landscapes of Tregs fundamentally differ across tumor types | Human (tumor-infiltrating, CRC) / Review | Moderate: Existing studies predominantly confined to single tumor types; systematic cross-tumor-type comparisons lacking | • No pan-cancer Treg single-cell multi-omics atlas available • No unified standards for cross-cancer Treg epigenetic analysis | ① Large-scale, multi-cancer, high-throughput, multi-omics Treg epigenetic atlas projects  ② Unified standards for cross-cancer Treg epigenetic analysis must be established |

**Part B: Target Safety Assessment for Precision Unlocking Strategies**

*( Cross-reference: Main text Section 8.1 "Surface Marker-Targeted Depletion" and Section 8.3 "Combination Strategies and Tumor-Type Specificity." Risk levels: ++ moderate; +++ high. See main text refs [109–139] for detailed clinical evidence)*

| Target | Primary Targeted Cells | Non-Tumor Tissue/Cell Expression | Potential Off-Target Toxicity | Risk Monitoring Methods | Risk Level |
| --- | --- | --- | --- | --- | --- |
| CCR8 | Tumor-infiltrating Tregs (high expression) (125,139,150) | • Th2 cells (IL-5-enriched subset) (132)  • Skin-resident T cells (132)  • Subset of dendritic cells (151)  • Dysfunctional CD4⁺ and CD8⁺ T cells within tumors | • Dermatitis, atopic dermatitis-like reactions  • Impaired type 2 immune responses  • Potential peripheral Treg depletion (CCR8⁺ Tregs present in blood and spleen) | • Skin examination (rash, dermatitis)  • Peripheral blood Treg immunophenotyping  • Long-term follow-up for autoimmune manifestations | ++ |
| CD25 (ADC) | Tumor-infiltrating Tregs (CD25-high expression) (134) | • Activated CD8⁺ T cells (intermediate CD25 expression) (134)  • Activated mast cells (CD25⁺ subset) (152)  • Subset of dendritic cells | • CD8⁺ Teff collateral damage via bystander effect  • Impaired immune activation  • Disruption of mast cell-related tissue homeostasis | • Peripheral CD8⁺ T cell count and function  • Serum tryptase (mast cell activation)  • Tumor biopsy: CD8⁺ T cell infiltration assessment | +++ |
| TIGIT | • Tumor Tregs (TIGIThigh CD226⁻ clonally expanded subset)(153)  • Exhausted CD8⁺ T cells  • Activated NK cells (154) | • NK cells (TIGIT modulates NK cytotoxicity; low TIGIT⁺ NK cells show enhanced function)(154)  • Intrahepatic CD56^bright NK cells (high TIGIT expression, involved in liver regeneration) (154)  • Tregs (TIGIT drives tissue repair factor Areg expression)(155) | • T cell overactivation (disinhibition)  • Impaired NK cell function  • Impaired liver tissue repair and regeneration  • Exacerbated tissue pathology during viral infections | • Liver function tests (ALT, AST)  • NK cell cytotoxicity assays  • Viral infection monitoring  • Cardiac biomarkers (troponin) for myocardial events | ++ |
| 4-1BB | Activated T cells (CD8⁺, CD4⁺), NK cells (156) | • Liver sinusoidal endothelial cells(157)  • Hepatic Kupffer cells (157)  • Activated endothelial cells and smooth muscle cells (157)  • Tumor vascular endothelial cells | • Dose-dependent hepatotoxicity (transaminitis)  • Vascular inflammation  • Endothelial cell activation-related toxicity | • Liver function tests (ALT, AST, bilirubin) at every cycle  • Imaging for vascular inflammation  • Dose-limiting toxicity protocols | +++ |
| OX40 | • Activated T cells (CD8⁺, CD4⁺)  • Tumor Tregs (high OX40 expression) (158) | • Activated effector T cells (dual role: Teff stimulation + Treg suppression)  • Potential compensatory Treg activation | • Treg expansion (compensatory)  • Cytokine release syndrome (CRS)  • Autoimmune-like reactions | • Peripheral Treg frequency monitoring  • CRS grading and management protocols  • Long-term autoimmune surveillance | ++ |

Table Note for Part B: Risk levels are assigned based on preclinical models, Phase I/II clinical data, and documented adverse events. ++ = moderate risk (manageable with standard monitoring protocols); +++ = high risk (significant safety constraint; necessitates built-in mitigation strategies such as affinity optimization, logic-gated targeting, or safety switches). For comprehensive clinical evidence and detailed risk-benefit analysis, refer to main text Sections 8.1–8.3. Abbreviations: ADC, antibody-drug conjugate; ADCC, antibody-dependent cellular cytotoxicity; ADCP, antibody-dependent cellular phagocytosis; ALT, alanine aminotransferase; AST, aspartate aminotransferase; CRS, cytokine release syndrome; Treg, regulatory T cell.
